# Physico-chemical properties based differential toxicity of graphene oxide/reduced graphene oxide in human lung cells mediated through oxidative stress

**DOI:** 10.1038/srep39548

**Published:** 2016-12-21

**Authors:** Sandeep Mittal, Veeresh Kumar, Nitesh Dhiman, Lalit Kumar Singh Chauhan, Renu Pasricha, Alok Kumar Pandey

**Affiliations:** 1 Academy of Scientific and Innovative Research (AcSIR), CSIR – IITR Campus, Lucknow, India; 2 Nanomaterials Toxicology Laboratory, Nanotherapeutics and Nanomaterial Toxicology Group, CSIR – Indian Institute of Toxicology Research (CSIR – IITR), Vishvigyan Bhawan, 31, Mahatma Gandhi Marg, PO Box – 80, Lucknow, Uttar Pradesh – 226001, India; 3 CSIR - National Physical Laboratory (CSIR-NPL), New Delhi - 110012, India; 4 Water Analysis Laboratory, Nanotherapeutics and Nanomaterial Toxicology Group, CSIR – Indian Institute of Toxicology Research (CSIR – IITR), Vishvigyan Bhawan, 31, Mahatma Gandhi Marg, PO Box – 80, Lucknow, Uttar Pradesh – 226001, India; 5 Electron Microscopy Laboratory, CSIR – Indian Institute of Toxicology Research (CSIR – IITR), Vishvigyan Bhawan, 31, Mahatma Gandhi Marg, PO Box – 80, Lucknow, Uttar Pradesh – 226001, India

## Abstract

Goraphene derivatives (GD) are currently being evaluated for technological and biomedical applications owing to their unique physico-chemical properties over other carbon allotrope such as carbon nanotubes (CNTs). But, the possible association of their properties with underlying *in vitro* effects have not fully examined. Here, we assessed the comparative interaction of three GD - graphene oxide (GO), thermally reduced GO (TRGO) and chemically reduced GO (CRGO), which significantly differ in their lateral size and functional groups density, with phenotypically different human lung cells; bronchial epithelial cells (BEAS-2B) and alveolar epithelial cells (A549). The cellular studies demonstrate that GD significantly ineternalize and induce oxidative stress mediated cytotoxicity in both cells. The toxicity intensity was in line with the reduced lateral size and increased functional groups revealed more toxicity potential of TRGO and GO respectively. Further, A549 cells showed more susceptibility than BEAS-2B which reflected cell type dependent differential cellular response. Molecular studies revealed that GD induced differential cell death mechanism which was efficiently prevented by their respective inhibitors. This is prior study to the best of our knowledge involving TRGO for its safety evaluation which provided invaluable information and new opportunities for GD based biomedical applications.

Carbon based nanomaterials (CBNMs) such as fullerene, carbon nanotubes (CNTs) and recently developed graphene have attracted substantial attraction of scientific community due to their wide applications in areas of biomedicine, industrial and nanodevices^1^. Among them, graphene is endowed with high surface to volume ratio, high mechanical strength, flexible nature and ease of functionalization over other carbon nanoform. Structurally, graphene is two dimensional honey comb lattice possessed single layer of carbon atoms and non-bonded π electron on above and below the layer^2^,^3^. Due to these remarkable properties, graphene since their discovery has sparked the vast interest in scientific and engineering community for numerous revolutionary applications.

Despite the potential of graphene in several applications, in unmodified state it suffers from poor dispersible nature, which makes the exploitation of its properties challenging and remain in its infancy^4^. To overcome this situation, one of the most successful approach is the use of graphene derivatives (GD) such as graphene oxide (GO) and reduced graphene oxide (rGO). Particularly, rGO is generally known as the product of GO reduction either through chemical (chemically reduced graphene oxide, CRGO), thermal (thermally reduced graphene oxide, TRGO) or electrochemical route. GO and rGO showed good dispersibility, stability in physiological environment and abundance of functional groups that opens up a plethora of potential applications exploiting their exciting properties in the field of nanoelectronics^5^, composite materials^6^, energy and storage technology^7^,^8^, bioimaging and biosensing^9^,^10^, drug delivery and cancer therapy^11^. Based on enormous research and development, 21^st^ century is considered as carbon age and recently GD have been reported to be used as nontoxic and implantable platform for growth of various cultured cells, in regenerative medicines and prosthetic applications^12^,^13^,^14^.

Before embarking on the potential use, a critical evaluation of the biological behavior of NMs is prerequisite to predict their undesirable responses^15^. Thus, with the expanded uses of GO and rGO, it is also essential to investigate their effects on human health and environment. Apart from, substantial literature on the exponential applications, a limited and moreover contrasting reports are available which represent the GD as a biocompatible material^16^,^17^ as well as shown to induce different type of pathology in *in vitro* and *in vivo* systems^18^,^19^,^20^,^21^,^22^. Based on above reports it is not possible to draw a conclusion regarding their biosafety and safer use in biomedical applications. This discrepancy could be due to the fact that the unique physico-chemical properties of NMs can actively interfere or manipulate biological systems as demonstrate for CNTs^23^. Thus being from the same family, it is also necessary to develop a hypothesis relating physico-chemical properties of GO and rGO to their biological responses. Particularly, there is dearth of information regarding how the lateral dimension and functional groups of different GD dictate their differential *in vitro* behavior and also the underlying molecular mechanism is still unknown. Such information is necessary to avoid generalization and description of all GD as being toxic to human and environmental health^24^.

Thus keeping in view the above scenario, in the present study, we have systematically investigated the *in vitro* effects of three GD – graphene oxide (GO), thermally reduced GO (TRGO) and chemically reduced GO (CRGO) in human lung alveolar adenocarcinoma cells (A549) and normal human lung bronchial epithelial cells (BEAS-2B) used as pulmonary like cell system. Since, NMs can enter into the body through various routes but the inhalation is most common route during manufacture and processing of NMs in occupational settings^25^. Thus, the lung cell line treated with GO and rGO may provide the probable scheme regarding occupational exposure of GD. A549 cells are characteristically type II pulmonary epithelial cells whereas BEAS-2B cells are responsible to maintain the mucosal integrity against various particulates. The main aim of this study was to identify the relationship between different physico-chemical properties of GD their induced *in vitro* effects. As a result we demonstrate a strong correlation between lateral size and functional group density and the ability of GD to interact with cells. Also, we demonstrate the activation of differential cell death pathway with the induction of necroptosis in cancer cells, as a function of severe stress, which can be harnessed to rationally design a nanocarrier for drug delivery and cancer therapy.

## Results and Discussion

### Synthesis and characterization

To assess the potential effects of GD on two different human lung cells, we have synthesized GO, CRGO and TRGO with varying physico-chemical properties, as shown in Supplementary Fig. S1.

**Figure 1:**
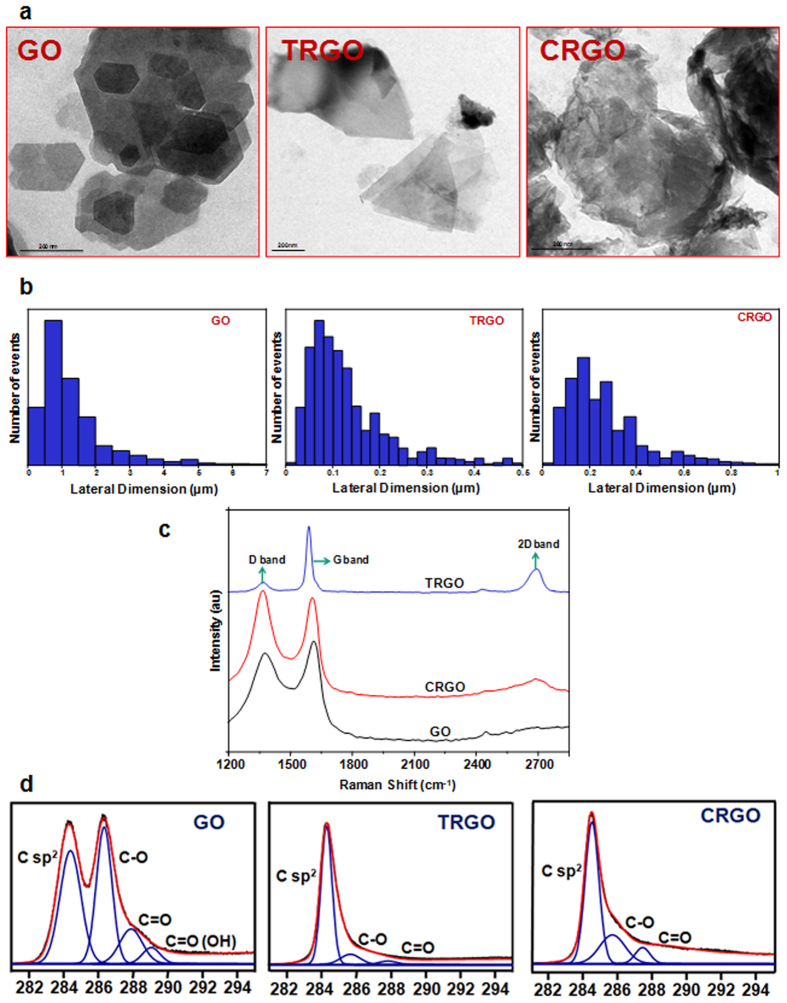
Physicochemical characterization of GD. TEM photomicrographs showing the (**a**) primary shape and (**b**) corresponding lateral size distribution of GO, TRGO, CRGO. (**c**) Raman spectra of GO, TRGO, CRGO indicate that these materials possess significant structural differences in graphitic planes with each other and rGO contains few layer graphene sheets. (**d**) The presence of functional groups and their percentage on the surface of GD was determined using XPS analysis which indicate the presence of oxygenated functional groups on the surface of GO which was eliminated after chemical and thermal reduction.

Since, the extensive characterization of NMs is of prime importance during their safety evaluation^26^ thus, we also employ a combination of analytical and spectroscopic methods to characterize our samples. The primary shapes of GO, CRGO, TRGO were observed from transmission electron microscopy (TEM) images as shown in Fig. 1a. The results revealed that all the samples were planar in shape and reduction of the GO further reduces the dimension of graphitic planes as shown for TRGO and CRGO (Fig. 1a).

Next, the lateral size distribution was obtained by performing a statistical analysis of several TEM images (Fig. 1b). The GO was found to contained large flakes with lateral dimension ranged from 0.2 μm to 0.6 μm. In contrast to this, TRGO and CRGO contained smaller sized flakes. Interestingly, TRGO was having smaller graphite plane ranged from 50 nm to 250 nm compared to CRGO (ranged from 100 nm to 400 nm) that could be due to the sudden exfoliation of graphite sheets and removal of functional groups at higher temperature. Since, for biomedical application the acceptable size limit for GO and rGO is ~50 nm to ~1000 nm and our samples were also in comparable lateral size range^27^.

It has been reported that NMs often form agglomerates in solutions which can affect their interaction with biological system. Thus, the characterization of our samples in liquid culture medium was carried out using dynamic light scattering (DLS). The hydrodynamic size and zeta potential as measured by DLS are presented in Supplementary Table S1. The DLS analysis showed that all three samples formed stable colloidal dispersion in culture medium compare to Milli-Q water which imparts their good dispersion which is of prime importance for biomedical applications.

The functional group density on the surface of GO, TRGO and CRGO was estimated using Fourier transform infrared (FTIR) spectroscopy as shown in Supplementary Fig. S2. The ATR FTIR Spectra of GO exhibited signature peaks at 3460 cm^−1^(ʋ_str._O-H), 1720 cm^−1^ (ʋ_str._C=O), 1627 cm^−1^ (ʋ_bend_ aromatic C=C, O-H), 1232 cm^−1^ and 1060 cm^−1^ (ʋ_str._ epoxy C-O and alkoxy C-O) which suggested the incorporation of oxygenated groups in the carbon skeleton during the oxidation of graphite. These groups render them hydrophilic in nature and allow further chemical modification useful for various biomedical applications. In contrast, after thermal and chemical reduction of GO most of the functional groups get removed. However, the reduction efficiency was more in TRGO compared to CRGO which showed the ability of thermal reduction to prepare graphene like sheets with high yield.

X-ray diffraction (XRD) spectroscopy is a prime analytical technique used for phase identification. The d_002_ value of bulk graphite used in earlier studies was found at 3.35 Å (2θ ≈ 26.54°) calculated by Bragg’s law. In GO, the value of d_002_ was increased from 3.35 Å to 8.42 Å (2θ ≈ 10.5°) which was due to the presence of oxygen containing functional groups (Supplementary Fig. S3). Further, in CRGO the d_002_ peak was found at ≈26.29° with broad Full Width at Half Maximum (FWHM). This signature d_002_ peak indicate the reduction of functional groups from GO and increased FWHM shows the reduced size of the graphene layers. In TRGO, the peak position (26.3 Å) was nearly same as that of CRGO, but FWHM was found to be reduced which indicates the higher degree of ordering as compared to CRGO (Supplementary Fig. S3).

Raman spectroscopy was utilized to characterize graphene structure, structural changes during reduction process and number of layers in GO, TRGO and CRGO (Fig. 1c). As synthesized GO showed two well defined characteristics peaks at 1345 cm^−1^ (D band) and 1595 cm^−1^ (G band) which corresponds to the structural imperfection, due to the attachment of functional groups and stretching of C-C bonds in graphitic materials^28^,^29^ respectively. Interestingly in GO, compared to pristine graphite the intensity of D band and G band was found to up shift which could be attributed to the activation and merging of the Raman inactive D’ band and exfoliation of graphite to single graphene sheets, consistent with the previous results^30^. In comparison, rGO showed more prominent D band with downshift of G band compared to GO which could be attributed to self healing of graphite consistent with the previous results^30^,^31^. In addition, TRGO showed a prominent 2D peak at around 2700 cm^−1^ which originates from a two phonons double resonance Raman process with very less intense D peak (Fig. 1c). Previously, it has been reported that by the analysis of shape, width and position of 2D peak, the layer number in graphene may be distinguished^32^. In our rGO sample, the position and shape of 2D peak suggest the formation of few layer (3 to 4 layer) graphene after the thermal and chemical reduction of GO. Furthermore, the Id/Ig ratio in GO spectrum is higher than CRGO confirming GO is the highly disordered structure because of the insertion of functionalities. For TRGO, very less intense D band was found in Raman spectrum pointing towards the relatively defect less structure. CRGO always have some residual functionalities as it does not get fully reduced, hence the presence of few defects (Fig. 1c).

Scanning electron microscopic images also elucidated the difference in the morphology of the tested GD. The GO was found to contain wrinkled surface and stacked sheet appearance compared to TRGO and CRGO (Supplementary Fig. S4). In contrast, well exfoliated and crumpled sheets were observed in rGO sample which is a common feature of the oxidation and reduction process^33^,^34^. The rGO samples (TRGO and CRGO) mainly consist of single or few layers as suggested by the SEM image (Supplementary Fig. S4). Moreover, sheets of micron sized lateral dimensions were present in CRGO sample which appeared to be wrinkled due to their thin structure and chemical tearing.

The X-ray photoelectron spectroscopy (XPS) analysis was carried out for the assessment of type and functional groups level on GD. The carbon to oxygen ratio (C/O) is a widely accepted criterion for the evaluation of reduction process. The XPS C1s core spectra was recorded for each sample which was fitted for the analysis of aromatic rings and hydrogenated carbon (C=C/C-C, C-H, 284.6–284.9 eV), epoxy groups (C-O-C, 286.9 eV), carbonyl groups (C=O, 288.2 eV) and carboxyl groups [C=O(OH), 289.3 eV]. Spectra showed a significant difference in the amount of above groups between three tested GD which analogized our FTIR results. According to the XPS survey scan, the C/O ratio was found to be in the order of TRGO > CRGO > GO which verified the successful reduction of GO with restoration of graphene structure, in particular TRGO sample (Supplementary Fig. S5). Further, C1s peak were deconvoluted using Gaussian-Lorentzian functions after Shirley background correction according to previous studies^33^. In GO sample, most of the carbon atoms found to have sp^3^ hybridization while in rGO most of the sp^2^ bonds recover. However, this recovery was more prominent in TRGO compared to CRGO which was in agreement to our Raman spectra showing the successful restoration of graphene structure after thermal reduction of GO (Fig. 1d).

In addition, atomic force microscopic (AFM) images showed a homogenous dispersion of each sample deposited by spin-coating of a dilute GD dispersion on silicon wafer (Supplementary Fig. S6). Similar to TEM, GO nano-sheets with lateral dimensions ranged from nm to hundred of micron were found to be distributed onto silicon surface. Thickness of GO sheet is given by height profile and found to be nearly 1 nm which was due to the presence of functional groups. In TRGO, the thickness of sheets was found to be nearly 0.5 nm whereas in CRGO it was 0.89 nm.

Taken together, these results demonstrated the successful synthesis of GO TRGO and CRGO with significant differences in their physico-chemical properties, especially in lateral size and functional groups density.

### Cytotoxic effect of GO, TRGO and CRGO in A549 and BEAS-2B cells

**Figure 2:**
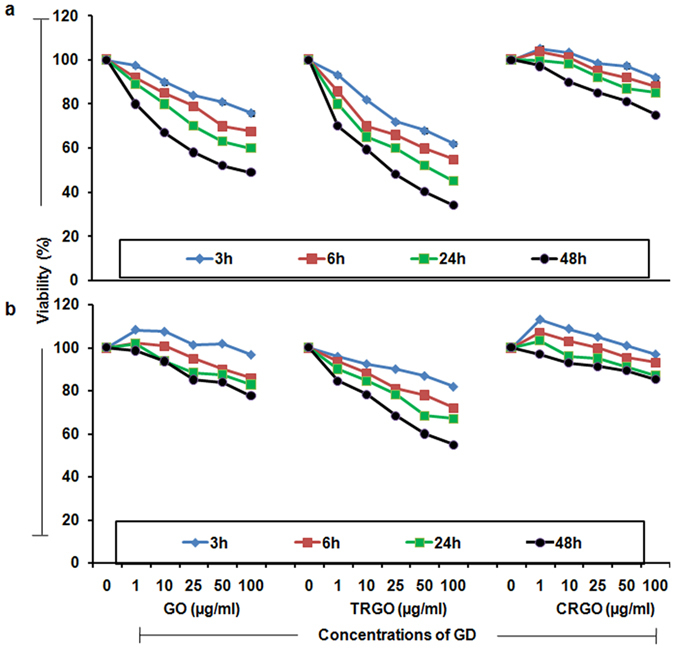
Effect of GD exposure on cellular viability of human lung cells. MTT assay was carried out for the assessment of reduction in viability of (**a**) A549 cells and (**b**) BEAS-2B cells incubated with GO, TRGO and CRGO for 3 h – 48 h time period at a concentration range of 1–100 μg/ml. Viability was significantly reduced in a dose and time dependent manner and the effects was more prominent in TRGO exposed and especially A549 cells. Data is mean ± SE of three independent experiment.

To observe the *in vitro* behaviour of GD, first of all in our study, A549 and BEAS-2B cells were exposed to 1–100 μg/ml concentrations and their cellular viability was determined using MTT assay over 48 h. The results demonstrated a significant reduction in viability of both cells upon GO and TRGO exposure with less effect in CRGO exposed cells which represents that these materials are toxic within the exposure range (Fig. 2). Interestingly, the effect was more pronounced in A549 cells (Fig. 2a) compared to BEAS-2B (Fig. 2b) cells upon exposure of GD. As expected this reduction was more prominent after 48 h exposure than the corresponding 24 h.

In our study, TRGO was found to induce more reduction in viability compared to GO and CRGO which is in agreement to the size dependent toxicity of CBNMs^34^. However, in such studies TRGO was not included than in our study thus aid more information regarding differential behaviour of GD. Since, GO contain more functional groups thus have a greater potential of cellular interaction compared to more biologically inert CRGO. Therefore, approximately same sizes GO is more toxic than CRGO which showed the role of functional groups towards biological interaction Overall, our cellular viability data demonstrated a different behaviour of GD based on physico-chemical properties with small lateral dimension and more functional groups induced more effects.

### ROS generation potential of GO, TRGO and CRGO

**Figure 3:**
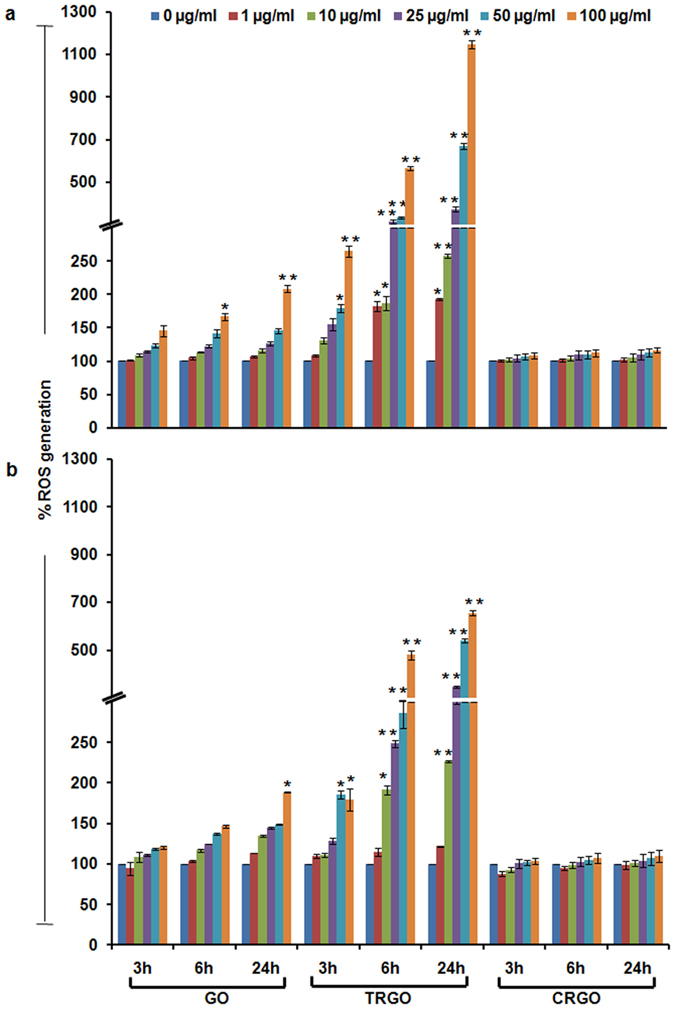
Effect of GD exposure on intracellular ROS generation in human lung cells. DCFDA assay was carried out for the assessment of ROS production in GO, TRGO and CRGO exposed (**a**) A549 cells and (**b**) BEAS-2B cells after 3 h – 24 h time period at a concentration range of 1–100 μg/ml which indicate a strong oxidative potential of TRGO compared to GO and CRGO. The A549 cells were found to be more affected by the ROS which subsequently deplete intracellular glutathione content compared to BEAS-2B cells. Data represents mean ± SE of three independent experiment. *p < 0.05, **p < 0.01, compared to control value.

As oxidative stress has been proposed as a key event in NMs induced toxicity^15^. Therefore to address the mechanism of cytotoxicity, we also assessed the oxidative stress potential of tested GD in A549 and BEAS-2B cells. Similar to the above viability results, the cellular oxidative stress manifested by elevated ROS levels and reduced GSH level was found to be significant in TRGO exposed and especially in A549 cells (Fig. 3 and Supplementary Fig. S7). In fact, CRGO leads to negligible alteration in ROS and cellular GSH level which could be the explanation of their less viability reduction potential. In addition, the inverse correlation between the viability decline and ROS elevation further indicated that oxidative stress was probably a key route by which GD induced damage to cells. Besides, in the presence of N-Acetyl-L-Cysteine (NAC) the ROS level was found to get attenuated in GD exposed cells. Taken together, these results suggested that the GO and TRGO might lead to the oxidative stress via the increased level of ROS production and decrease in antioxidant enzymes levels by the cell self-protection.

### Genotoxicity potential of GO, TRGO and CRGO

**Figure 4:**
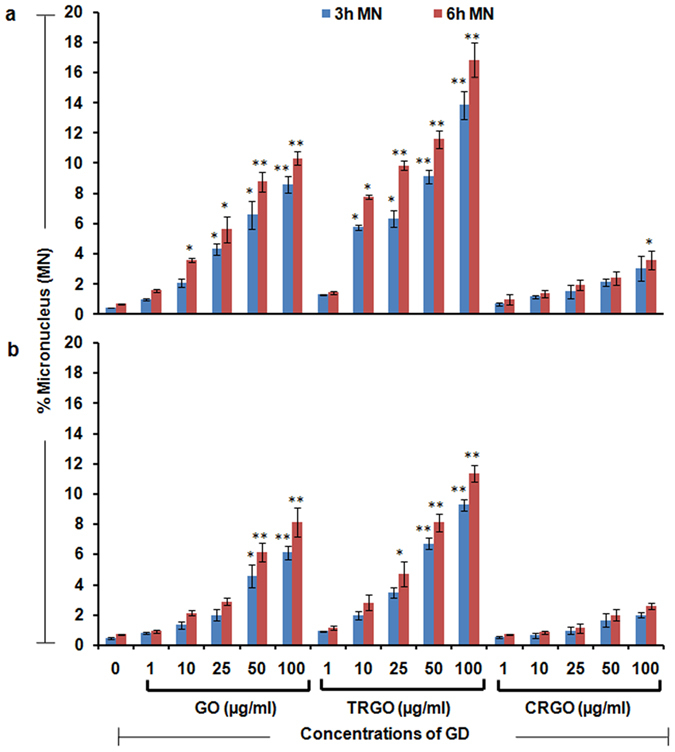
Genotoxicity potential of GD in human lung cells. Flow cytometry based micronucleus (MN) assay indicate a significant increase in MN upon the exposure of GO, TRGO and CRGO in (**a**) A549 cells and (**b**) BEAS-2B cells after 3 h and 6 h time period at a concentration range of 1–100 μg/ml. A notable effect was found in TRGO exposed and more especially A549 cells which showed the DNA damaging potential of GD. Data represents mean ± SE of three independent experiment. *p < 0.05, **p < 0.01, compared to control value.

It has been reported that excessive production of ROS can induce genomic instability which may further affect the cellular homeostasis by inducing mutation, tumorigenesis and subsequently cell death^35^. Thus, due to the presence of excessive ROS in our study we also assessed the micronucleus (MN) formation in GO, TRGO and CRGO exposed cells by flow cytometry based method after 3 h and 6 h of exposure. A significant increase in MN number was found after GO and TRGO exposure in both cells as shown in Fig. 4. Similarly to the previous results, CRGO induce negligible increase in MN number and A549 cells showed more pronounced effect compared to BEAS-2B (Fig. 4, upper panel). In particular, TRGO was found to exert more effect which suggests that excessive ROS production in TRGO exposed cells lead to more DNA damage compared to other two GD. Our results are in accordance to the previous results which showed the genotoxicity potential of CBNMs^36^,^37^. These observations further support our viability and oxidative stress data which showing the increased alteration in cellular processes of TRGO exposed cells compared to GO and CRGO.

### Analysis of cell death mode in GO, TRGO and CRGO exposed cells

**Figure 5:**
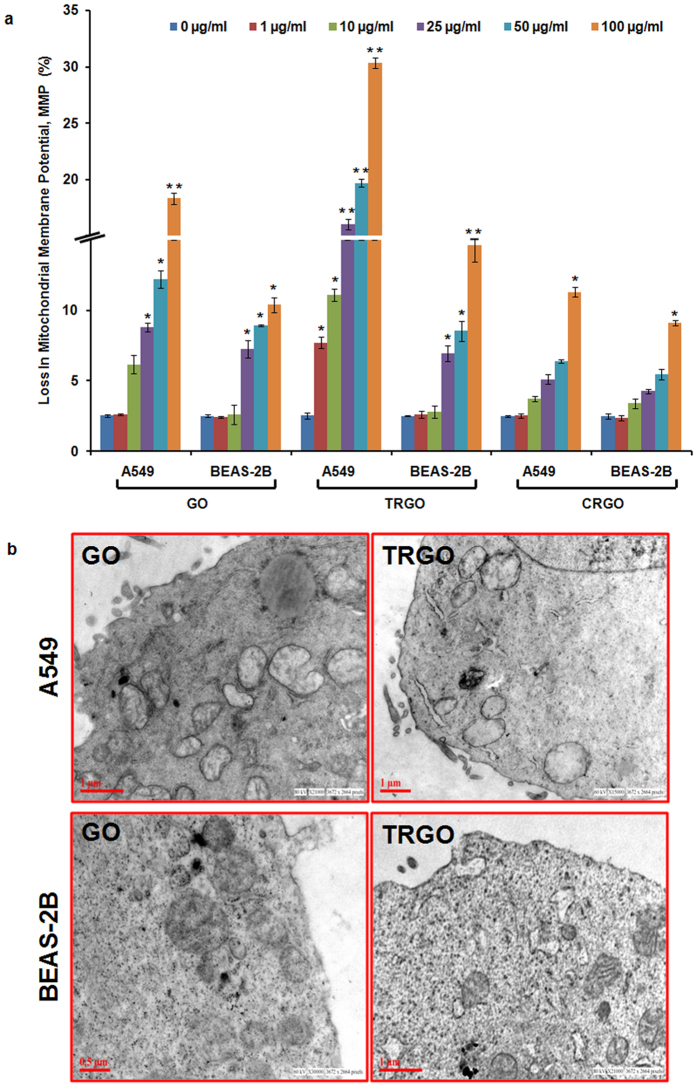
Effect of GD on mitochondrial homeostasis of human lung cells. (**a**) Flow cytometry based JC-1 assay showed a significant reduction in MMP of A549 and BEAS-2B cells after 24 h exposure of GO, TRGO and CRGO. (**b**) Representative TEM photomicrographs also showed the damage to mitochondrial cristae structure in both cells. Data is mean ± SE of three independent experiment. *p < 0.05, **p < 0.01, compared to control value.

It has been evident that induction of oxidative stress by NMs may lead to cell death^15^,^38^,^39^,^40^ via the activation of inflammation signalling and cell death pathways^41^,^42^. ROS are highly reactive which can damage the cellular vital organs. Importantly, any impairment in mitochondrial functions could also significantly enhance the amount of ROS thus the effect of GD on mitochondrial integrity was investigated using JC-1 dye and TEM analysis. Consistently with the above results, a significant decrease in green to red fluorescence intensity (marker of decline in mitochondrial membrane potential) was found in both cells (Fig. 5a). In particular, TRGO induced more decline especially in A549 cells (Fig. 5a, upper panel). In addition, TEM analysis also revealed swollen and hollow mitochondria in GO and TRGO exposed cells with more effect in TRGO exposed A549 cells compared to elongated mitochondria in control cells showing the alteration of mitochondrial homeostasis upon exposure to GD (Fig. 5b).

**Figure 6:**
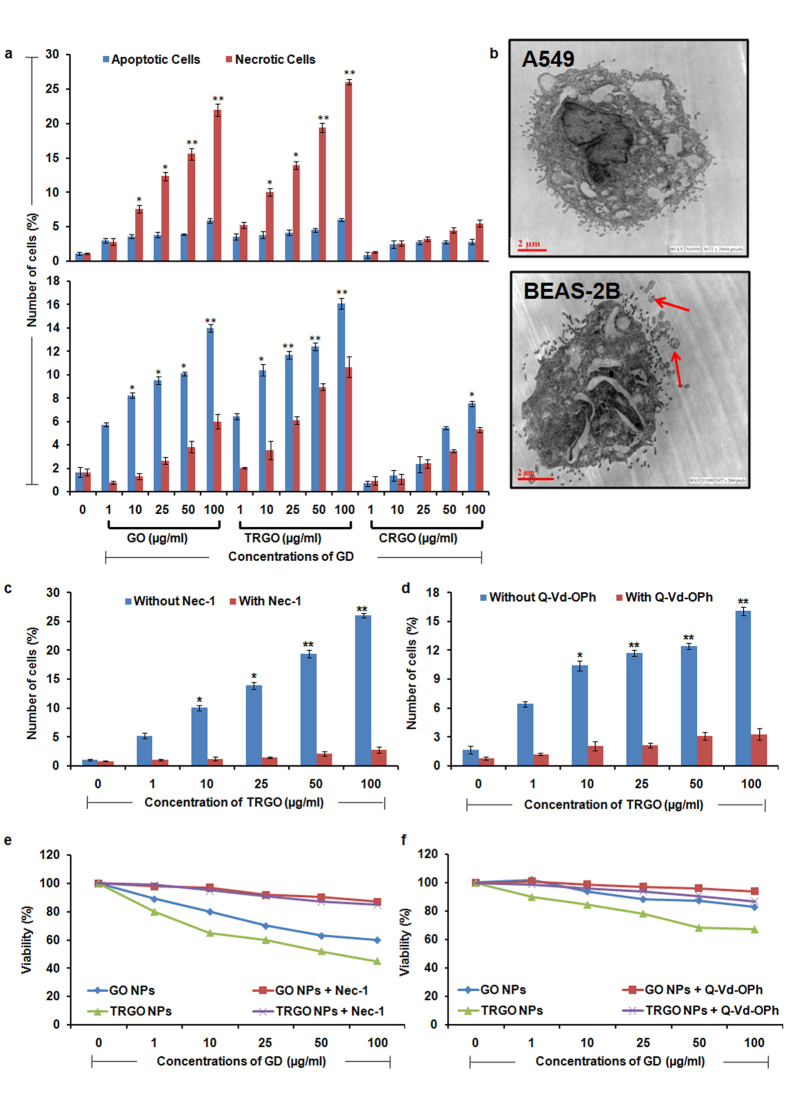
Mode of cell death analysis in GD exposed human lung cells. (**a**) Flow cytometry based Annexin V/PI assay showed a significant increase in necrotic cells in A549 (upper panel) whereas apoptotic cells increase in BEAS-2B (lower panel) after 24 h exposure. (**b**) Representative TEM photomicrographs showing necrosis in A549 and apoptosis in BEAS-2B cells (red arrow – apoptotic bodies). (**c**) Effect of respective inhibitor i.e. Nec-1 (necroptosis inhibitor) on necrotic cell death in A549 cells whereas Q-vd-Oph (apoptosis inhibitor) on apoptosis in A549 cells (**d**) was also evaluated. Both inhibitors was found to alter the expression level of respective genes which indicate the activation of differential cell death pathway in GD exposed cells which depends upon the cellular phenotype. Further, effect of respective inhibitors on cell death in A549 (**e**) and BEAS-2B cells (**f**) after exposure of GO and TRGO denotes the rescue of both cells and restoration of cellular viability. Data represents mean ± SE of three independent experiment. *p < 0.05, **p < 0.01, compared to control value.

Further, the Annexin V/PI staining explicitly demonstrated the induction of differential cell death pathway depending upon the cellular phenotype. Interestingly, there was a significant increase in necrotic cell population (Annexin V^−^/PI^+^ cells) in A549 cells (Fig. 6a, upper panel) whereas BEAS-2B cells showed increase in apoptotic cell population (Annexin V^+^/PI^−^) upon exposure to GO and TRGO (Fig. 6a, lower panel). In accordance to the above viability and oxidative stress results, the observed effect was more pronounced in TRGO exposed cells and is well correlated with small size and functional groups density on the GD surface. In addition to this, TEM photomicrographs further confirms the differential cell death activation in A549 as well as BEAS-2B upon the exposure of GD (Fig. 6b). Taken together the results on viability, oxidative stress and cell death pathway, it could be hypothesized that the GD induced adverse effects is largely depend upon the physico-chemical properties such as lateral dimension, functional groups, sharp edges which induced distinct cell death mechanism.

In the recent studies it has been prove that necrosis can also be a regulated form of cell death as apoptosis which can be assessed by the use of Nec-1^43^,^44^. Interestingly here, the application of Nec-1 significantly decrease the necrotic cells population in TRGO exposed A549 cells suggesting the activation of regulated necrosis (Fig. 6c). However Nec-1 does not shown any effects towards the cell death induced in BEAS-2B cells. Further, cell death in BEAS-2B was significantly reversed by the use of Q-Vd-Oph (a potent inhibitor of caspases) which proved the involvement of caspases induced apoptotic cell death in BEAS-2B cells (Fig. 6d).

Next, the activation of differential cell death was also confirmed by assessing the effect of Nec-1 and Q-Vd-Oph on cellular viability of A549 and BEAS-2B, respectively. A significant attenuation in the viability reduction was found with the pre treatment of Nec-1 to A549 (Fig. 6e) whereas Q-Vd-Oph to BEAS-2B (Fig. 6f) prior to GO and TRGO exposure. Thus the combined results showed the activation of differential cell death pathway after exposure of GD which largely depends upon the cell type used.

### Analysis of cell interaction with GO and TRGO

Since significant differences have been evidenced between A549 and BEAS-2B cells responses to our GD samples, including the diverse impact of GO, TRGO on cellular viability, oxidative stress generation and cell death. These effects could be accounted for their differential interaction with cells and to possibly identify the underlying mechanism, we studied the interaction of cells with GD and their subsequent cellular internalization using flow cytometry and TEM.

**Figure 7:**
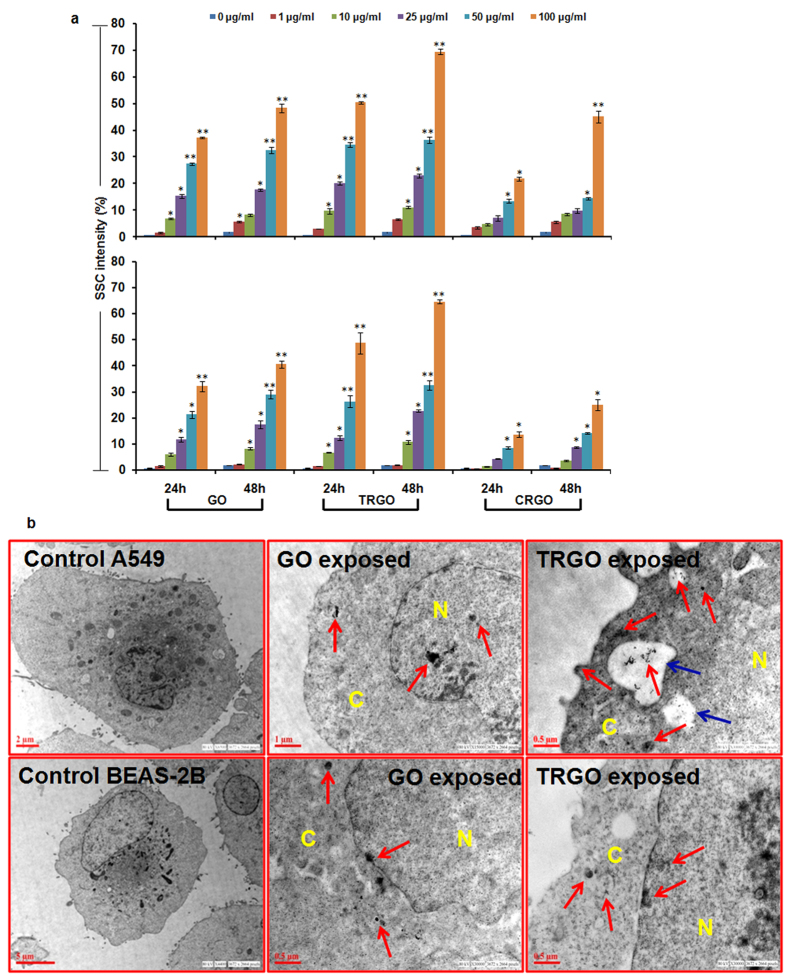
Cellular internalization assessment of GD in human lung cells. (**a**) Flow cytometry based analysis showed a significant increase in SSC intensity of A549 cells (upper panel) as well as BEAS-2B cells (lower panel) with more prominent effect in TRGO exposed cells. (**b**) Representative TEM photomicrographs showing the accumulation of GO and TRGO in cytoplasm (denoted by C) as well as in nucleus (denoted by N) with induction of vacuolization (blue arrow) in TRGO exposed A549 cells. Red arrow showing the presence of GD inside the cells. Data represents mean ± SE of three independent experiment. *p < 0.05, **p < 0.01, compared to control value.

To address this issue, first of all the flow cytometry based cellular uptake analysis of GO, TRGO and CRGO in both cells was carried out. A significant concentration and time dependent increase in the SSC intensity (marker of cellular granularity) of both cells exposed to GO and TRGO was found (Fig. 7a). In particular, TRGO significantly internalized more as compared to the other two GD in both cells. The order of GD internalization was TRGO > GO > CRGO and A549 > BEAS-2B (Fig. 7a). This difference in internalization can be attributed to the different physico-chemical properties of GD and different characteristic of cancerous cells than normal cells. Previously, it has been reported that GD mainly enters into the cells by endocytosis/phagocytosis depending upon their interaction with cellular membrane^45^. Since the phagocytic activity of cancer cells (A549) is greater than normal cells (BEAS-2B) and TRGO have small lateral dimension and more sharp corners than CRGO and GO. Thus, the observed more internalization in A549 cells and in particular TRGO exposed cells could be attributed to the above properties (Fig. 7a, upper panel). Further, GO compared to CRGO have more functional groups which could provide them a greater potential to interact with cellular membrane thus leading to increased internalization than CRGO.

In addition, TEM analysis also demonstrates the presence of GO and TRGO inside both cells (Fig. 7b). In particular, TRGO was found to be packed inside endocytic/phagocytic vesicles compared to GO. Interestingly, these vesicles were more prominent in A549 compared to BEAS-2B cells which could be due to more phagocytic activity of A549 cells. Further, there was more obvious disruption of cytoplasm structure in A549 compared to BEAS-2B cells with the damage to cellular membrane. Including this, both materials were found to be present inside the cytoplasm, mitochondria and more importantly in the nucleus which could be attributed to their genotoxicity potential (Fig. 7b).

In the milieu of the possible internalization mechanism identification, TEM analysis suggest the involvement of phagocytic internalization pathway for TRGO whereas passive disruption mechanism for GO. Owing to the small size, TRGO was efficiently taken up by the cells which caused the formation of vesicle whereas due to large size, GO parallel arranged to the cellular membrane, pierce it and then enter into the intracellular compartment (Supplementary Fig. S9). Our results are in accordance to the previous computer simulation study involving artificial membrane which suggested the translocation of large graphene sheets inside membrane via penetration compared to small sheets^46^. Including this, the presences of negatively charged groups on the surface of GO provide a strong electrostatic interaction between GO sheets and membrane leading to close interaction and provoke a physical damage which could be accounted for their presence in the cell cytoplasm^47^,^48^.

These results together suggest the role of lateral dimension, functional groups as well as cellular phenotypes towards differential uptake of GD. Thus considering these effects, the increased effect of TRGO on cellular viability, oxidative stress, genotoxicity and cell death could be accounted for their more internalization compared to GO and CRGO.

### Molecular analysis of cell death pathway in GD exposed cells

**Figure 8:**
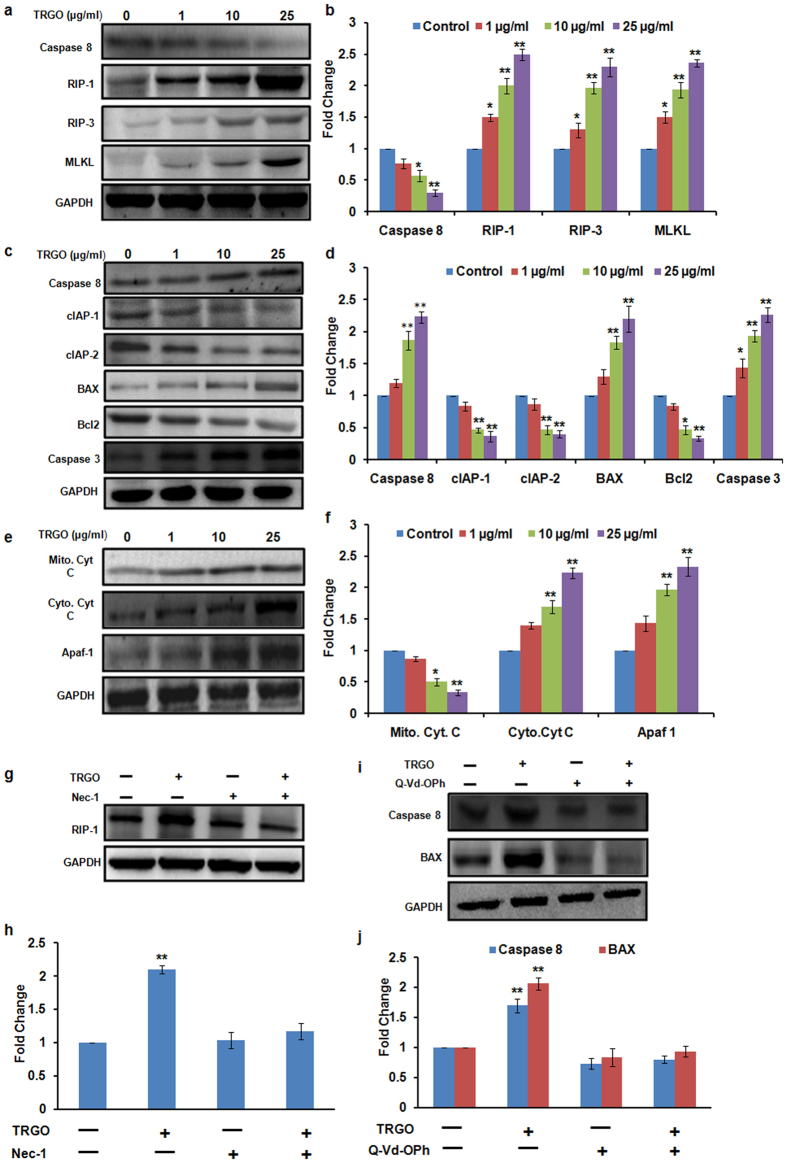
Western blot analysis of GD exposed cells for the evaluation of cell death pathway. A significant increase in the expression level of necroptotic protein in A549 cells (**a**) whereas increase in apoptotic protein in BEAS-2B cells (**c,e**) was found after the 24 h exposure of TRGO. (**b,d,f**) Quantitation was done in Biorad Versa DOC (Bio-Rad Laboratories, Inc. Hercules, USA) with the help of Quantity One Quantitation Software version 4.3.1 and expressed in fold change. Further, in the presence of Nec-1 (**g**) and Q-Vd-OPh (**i**) the expression level of respective proteins was found to get attenuated which confirmed the activation of differential cell death pathway upon GD exposure. (**h,j**) Quantitation was done in Biorad Versa DOC (Bio-Rad Laboratories, Inc. Hercules, USA) with the help of Quantity One Quantitation Software version 4.3.1 and expressed in fold change. Full lengths blots were shown in Fig. S11.

The molecular machinery of apoptosis has been widely explored whereas the underlying mechanism of necroptosis remains poorly understood. Recent studies have shown that receptor interaction protein kinase-1 (RIP-1), receptor interaction protein kinase-3 (RIP-3) and mixed lineage kinase linkage (MLKL) played a crucial role in the induction of necroptosis^49^,^50^. Thus, in order to determine the specific pathway, western blot analysis of various apoptotic and necroptotic proteins in GD exposed A549 and BEAS-2B cells lysate was carried out. As shown in Fig. 8, there was a significant increase in RIP-1, RIP-3 and MLKL proteins level showing the induction of necroptotic cell death pathway in A549 cells (Fig. 8a) whereas in BEAS-2B cells there was a significant increase in BAX: Bcl-2 ratio, cleaved caspase 3, cytosolic Cyt. C and Apaf-1 (Fig. 8c,e) after 24 h exposure which confirms the activation of apoptosis. However, in presence of respective inhibitors i.e. Nec-1 (necroptosis) and Q-Vd-Oph (apoptosis), proteins level related to the specific pathways were found to be altered (Fig. 8g,i). Thus these results confirm the involvement of differential cell death pathways in TRGO exposed BEAS-2B and A549 cells. Further, caspase 8 was found to play a central role in mediating the differential cell death pathway. As shown in Fig. 8a the expression level of caspase 8 was found to be down regulated in A549 cells which further increased the expression level of necroptosis protein. In contrast to this, the expression level of caspase 8 in BEAS-2B was found to up regulate (Fig. 8c) which simultaneously increases the expression level of pro-apoptotic protein after 24 h exposure of TRGO.

### Role of oxidative stress in GD induced cellular demise

Recent studies have shown that over production of ROS due to NMs exposure can leads to initiation of cell death pathways in the exposed cells^15^,^39^. Therefore, we also explored the possible role of ROS in GD induced cell death by the application of NAC. It was found that NAC have significant effect over the expression level of various apoptotic and necroptotic cell death pathways proteins (Supplementary Fig. S10a). Including this, NAC also attenuate the viability reduction after the GD exposure in A549 and BEAS-2B cells (Supplementary Fig. S10c). Taken together, these data clearly indicate that ROS generation plays an important role in the induction of apoptotic and necroptotic cell death pathways in A549 and BEAS-2B cells respectively after the exposure of GD.

## Conclusion

In this study we highlight the role of physico-chemical properties of GD towards their differential *in vitro* behaviour in human lung (normal as well as cancer) cells. It is of paramount interest to assess such effect to avoid the generalization in GD based research. We demonstrate that the lateral size and functional groups density is very likely responsible for the differential behaviour of GD in human lung cells. The effects observed suggest that TRGO had major effect on cellular viability, oxidative stress, genotoxicity and cell death compared to CRGO and GO owing to its small lateral dimension, sharp corners which facilitate its increased cellular uptake. Including this, the presence of functional groups on GO provides them a major affinity towards cell membrane which lead to increased effect than CRGO. Along this, A549 cells are found to be more susceptible to these changes in cellular machinery compared to BEAS-2B cells with differential cell death mechanism. The observed changes in cell machinery were also found to be correlated with the overproduction of ROS in both cell lines.

The study underscores the importance of physico-chemical properties characterization especially lateral size and functional groups of GD during their bioactivity assessment. Since the adverse effect of TRGO was not studied till now thus our study adds more knowledge about the relationship between physico-chemical properties of GD with their adverse effects. Also, the different behaviour of GD with two type of cells further suggest the tuning and exploitation of such properties for new therapeutic strategies based on specific cellular interactions. However, further studies with the incorporation of more cell lines are necessary to confirm this scenario. In particular in our study, necroptosis was found in cancer cells with the activation of apoptosis in normal cells which could be the result of excessive stress induced by GD in cancer cells. This phenomenon can be used to overcome the cancer cells ability to evade programmed cell death and can open the new way for graphene based cancer therapy.

## Methods

### Synthesis of GO, TRGO and CRGO

Graphene Oxide (GO): GO was prepared by modified Hummers method^51^. In this process, graphite powder (1 g) and sodium nitrate (NaNO_3,_ 1 g) were dissolved in H_2_SO_4_ (46 ml) in an ice bath. Thereafter, KMnO_4_ (6 g) flakes was slowly added to the above mixture under stirring until this solution becomes dark green. The solution was then transferred to 35 ± 5 °C water bath and kept on vigorous stirring for about an hour till the consistency resembles a thick paste. Further, Milli-Q water (96 ml) was added, and the solution was stirred for 30 min at 90 ± 5 °C. To this solution, Milli-Q water (200 ml) was added followed by the slow addition of H_2_O_2_ (30%, 6 ml), turning the colour of the solution from dark brown to yellow. The warm solution was then filtered and washed with Milli-Q (200 ml) water. The filter cake was thereafter dispersed in Milli-Q water by mechanical agitation and centrifuged for 3–5 times at low (2000 rpm) & high (8000 rpm) speed for 2 min & 15 min respectively and repeated until the supernatant attains neutral pH. The final sediment was termed as GO.

Thermally reduced GO (TRGO): As prepared GO was subjected to thermal treatment at 1000 °C for 10 s to give TRGO.

Chemically reduced GO (CRGO): The chemical reduction of GO to make CRGO was done by mixing aqueous solution of GO and hydrazine hydrate in ratio 10:7 under stirring condition. The mixture was kept for 1 h at 95 °C to produce the CRGO.

### Characterization of GO, TRGO and CRGO

Preparation of suspension: For the characterization purpose, stock suspensions (150 μg/ml) of GO, TRGO, CRGO were prepared by re-suspending them in Milli-Q, DMEM F-12 and RPMI culture medium. To reduce the agglomeration, suspension was subjected to probe sonication (Sonics Vibra cell, Sonics & Material Inc., New Town, CT, USA) at 30 W for total 10 min (2.5 min pulse on and 1 min pulse off for 4 times) and allowed to cool. Further different concentrations ranging from 1–100 μg/ml were prepared using the stock solution (150 μg ml^−1^).

Transmission electron microscopy (TEM): Samples for TEM analysis were prepared by drop-coating of GO, TRGO and CRGO at a concentration of 50 μg/ml on carbon-coated copper TEM grids. The grids were allowed to dry prior to measurement. TEM measurements were performed at an accelerating voltage of 120 kV on a Tecnai^TM^ G2 spirit (FEI, Netherland) instrument.

Dynamic Light Scattering (DLS): The average hydrodynamic size, size distribution and zeta potential of GO, TRGO and CRGO in solutions were determined by dynamic light scattering and phase analysis light scattering respectively using a Zetasizer Nano-ZS equipped with 4.0 mW, 633 nm laser (Model ZEN3600, Malvern instruments Ltd., Malvern, UK).

Fourier Transform Infrared Spectroscopy (FTIR): FTIR analysis of GD has been performed with the scan range 400–3800 cm^−1^ at the resolution of 8 cm^−1^ using Nicolet 5700 FTIR spectrometer.

X-Ray diffraction analysis (XRD): Powder X-ray diffraction of GO, TRGO and CRGO has been done using Bruker D8 Advance X-Ray diffractometer using Cu Kα radiation (λ = 1.541 A°).

Raman spectroscopy: Raman spectra were recorded by using Raman spectrophotometer (EVA) at a wavelength of 514 nm.

Scanning electron microscopy: SEM analysis was carried out for the surface morphological analysis of GO, TRGO and CRGO. Briefly, a small amount of powder sample was put on carbon tape, sputter coated with gold and visualized at an accelerating voltage of 30 kV on a FEI Quanta FEG 450 field emission scanning electron microscope with EDAX (FEI, Netherland) instrument.

Atomic Force Microscopy (AFM): AFM analysis was carried out to assess the size and thickness of GO, TRGO and CRGO. Briefly, Aqueous solution of GD was spin coated on nearly 12 mm square surface of argon plasma cleaned silicon dioxide. Imaging was performed using AFM Multimode-V, Veeco in tapping mode.

X-ray photoelectron spectroscopy (XPS): XPS measurements were performed using PHI 5000 Versa Prob II, FEI Inc. spectrometer using nonmonochromatic Al Kα radiation (1486.6 eV). XPSPEAK41 software with a Gaussian−Lorentzian line shape was used for the deconvolution of individual spectral peaks.

### Cell culture and exposure

Human lung alveolar adenocarcinoma (A549) cells and normal human lung bronchial epithelial (BEAS-2B) cells were used in this study as *in vitro* models. A549 cells were procured from American type cell culture (ATCC), Carlsbad, USA and cultured in DMEM F-12 culture medium (Invitrogen, Carlsbad, CA, USA), supplemented with 10% heat in-activated foetal bovine serum (FBS), 0.2% sodium bicarbonate and antibiotic antimycotic solution (10 ml/L), at 37 °C under a humidified atmosphere of 5% CO_2_.

BEAS-2B cells were also obtained from American type cell culture (ATCC), Carlsbad, USA and maintained in BEGM bullet kit (Lonza Clonetics Corporation, Switzerland). Prior to 24 h of treatment, BEAS-2B cells were cultured in RPMI-1640 medium supplemented with 10% FBS, 0.2% sodium bicarbonate and antibiotic antimycotic solution (10 ml/L) at 37 °C under a humidified atmosphere of 5% CO_2_.

At 80–90% confluency, both cells were harvested using 0.25% trypsin-EDTA solution and were sub cultured into 96 well plate, 12 well plate, 6 well plates, and 75 cm^2^ culture flask according to the requirement of experiment. Prior to treatment, cells were allowed to attach the culture surface for 22 h for A549 and 24 h for BEAS-2B and then exposed to varying concentrations (1–100 μg/ml) of GD (as prepared in preparation section) for different time points according to the need of experiment. In each experiment cells without nanoparticles were used as a control.

### Cytotoxicity assessment

MTT assay: Viability of A549 and BEAS-2B cells exposed to GO, TRGO and CRGO for the time period of 3 h–48 h were evaluated by MTT (3-[4,5-dimethylthiazol-2-yl]-2,5-diphenyltetrazolium bromide) assay according to the defined protocol^52^. Briefly, cells at a density of 10^4^ cells/100 μl/well were cultured in 96 well plate and exposed to 1–100 μg/ml concentration of GD for indicated time period. After exposure, cells were incubated with MTT dye for 3 h and resultant formazan crystals were dissolved in DMSO. Absorbance was taken at 570 nm using SYNERGY-HT multiwell plate reader, Bio-Tek (USA) with KC-4 software.

### Cellular internalization assessment

Flow cytometry based cellular uptake analysis: Flow cytometry based GD internalization assessment in cultured cells was carried out according to the defined protocol^53^. According to this method, increase in the intensity of side scattered (SSC) light with constant intensity of forward scattered (FSC) light in exposed cells is the remark of cellular uptake of NMs in cell. In brief, 1 × 10^5^ cells/ml/well were seeded in 12 well culture plate and exposed to varying concentrations (1–100 μg/ml) of GD for 24 h and 48 h. After completion of exposure time, cells were harvested using 0.25% trypsin and centrifuged at 1000 rpm for 10 min. Supernatant was discarded and the pellet was re-suspended in 1X PBS (500 μl). Acquisition and analysis was made using flow cytometer equipped with 488 nm laser (FACS Canto^TM^ II, BD Biosciences, San Jose, CA, USA) instrument.

TEM analysis: Ultrathin section of cells exposed to different GD (GO, TRGO and CRGO) were analysed using TEM for cellular internalization. Briefly, cells were fixed, embedded and thin sections were cut, stained, analysed using Tecnai^TM^ G2 spirit (FEI, Netherland) instrument at an accelerating voltage of 80 kV.

### Measurement of intracellular reactive oxygen species generation

The intracellular ROS generation was measured by method of Wan *et al*.^54^ and modified by Wilson *et al*.^55^ using 2, 7 dichlorofluorescein diacetate (DCFDA) dye. Briefly, cells (1 × 10^4^ cells/100 μl/well) were seeded in 96-well black bottom plate and exposed to varying concentration (1–100 μg/ml) of GD for 3 h, 6 h and 24 h. After completion of exposure time, medium was aspirated and cells were washed twice with cold 1X PBS. Thereafter, 1X PBS (100 μl) containing DCFDA dye (20 μM) was added to each well. The plate was incubated for 30 min at 37 °C and then DCFDA was discarded. Then 1X PBS (200 μl) was added to each well and fluorescence intensity was measured in a SYNERGY-HT multiwell plate reader, Bio-Tek, USA using KC-4 software at excitation and emission wavelengths of 485 nm and 528 nm, respectively.

### Oxidative stress markers analysis

Cells were cultured in T-75 cm^2^ culture flasks at a final density of 6 × 10^6^ cells/flask and exposed to the GO, TRGO and CRGO for 3 h and 6 h. After exposure, the cells were washed twice and then scrapped on ice using 1X PBS. The cells were centrifuged at 1200 rpm for 10 min and the pellet was re-suspended in cell lysis buffer to obtain cell lysate. Protein content was measured by Bradford method^56^ using BSA as standard.

Glutathione measurement: GSH content was measured in lysate according to the method of Ellman^57^ and expressed as μmole/mg of protein.

### Mitochondrial membrane potential (MMP) analysis using lipophilic fluorochrome

For the MMP analysis, fluorescence intensity of lipophilic cationic JC-1 (5,5′, 6,6′-tetrachloro-1, 1′3′3′-tetraethylbenzimidazolecarbocyanine iodide) dye was used as a reporter. This dye has dual fluorescence nature and upon induction of loss in MMP, fluorescence of this dye changes from red to green. Briefly, 1 × 10^5^ cells/well were exposed to 1–100 μg/ml concentration of GD for 24 h, washed with 1X PBS and incubated with JC-1 (10 μM) dye in culture medium for 15 min at 37 °C. Then cells were again washed and re-suspended in 1X PBS (400 μl). The cells were analyzed for red and green fluorescence in a BD FACS Canto II flow cytometer (BD Biosciences, San Jose, CA, USA) coupled with 485 nm wavelength excitation filter and 590 nm wavelength emission filters.

### Determination of mode of cell death (apoptosis/necrosis) in exposed cells

Apoptosis kit (FITC Annexin V Apoptosis detection Kit, BD Biosciences, USA) was employed to detect apoptotic and necrotic cells after exposure of GO, TRGO and CRGO. The manual of the kit was strictly followed. Briefly, 1 × 10^5^ cells/ml/well were plated in the 12-well culture plate and incubated with different concentrations (1–100 μg/ml) of GD for 24 h. Upon completion of exposure time, cells were harvested and washed twice with cold 1X PBS, re-suspended in 1X binding buffer (0.1 ml) containing FITC-Annexin V (5 μl) and PI (5 μl) and kept at room temperature in dark. After 10 min of incubation, 1X binding buffer (0.4 ml) was further added to each sample and analyzed using flow cytometer (FACS Canto^TM^ II, BD BioSciences, San Jose, CA, USA).

### Cell cycle progression analysis

A549 and BEAS-2B cells treated with different concentrations (1–100 μg/ml) of GO, TRGO and CRGO for 24 h were subjected to flow cytometry for the analysis of cell cycle perturbation upon exposure. Detailed protocol for sample preparation and analysis are given in the Supplementary information (SI method section 1).

### Genotoxicity potential of GO, TRGO and CRGO

Flow Cytometric analysis of micronucleus formation (MN) in GO, TRGO and CRGO exposed A549 and BEAS-2B cells was done according to the previous method^58^. Detailed protocol for sample preparation and analysis are given in the Supplementary information (SI method section 2).

### Western Blot Analysis

A549 and BEAS-2B cells were cultured and exposed to 1–100 μg/ml concentrations of GO, TRGO and CRGO for 24 h time period. After completion, cellular lysate was subjected to SDS PAGE for the expression level analysis of various protein related to necroptosis and apoptosis. Detailed protocol for sample preparation and analysis are given in the Supplementary information (SI method section 3).

### Statistical analysis

Results were expressed as mean ± SEM of three independent experiments and data were analyzed using one way analysis of variance (ANOVA) with Dunnet post hoc test to determine significance relative to control. In all cases, p < 0.05 was considered significant.

## Additional Information

**How to cite this article**: Mittal, S. *et al*. Physico-chemical properties based differential toxicity of graphene oxide/reduced graphene oxide in human lung cells mediated through oxidative stress. *Sci. Rep.* **6**, 39548; doi: 10.1038/srep39548 (2016).

**Publisher's note:** Springer Nature remains neutral with regard to jurisdictional claims in published maps and institutional affiliations.

## Supplementary Material

Supplementary Information
