# Erratum: Physico-chemical properties based differential toxicity of graphene oxide/reduced graphene oxide in human lung cells mediated through oxidative stress

**DOI:** 10.1038/srep41200

**Published:** 2017-01-20

**Authors:** Sandeep Mittal, Veeresh Kumar, Nitesh Dhiman, Lalit Kumar Singh Chauhan, Renu Pasricha, Alok Kumar Pandey

Scientific Reports 6: Article number: 3954810.1038/srep39548; published online: 12 21 2016; updated: 01 20 2017

The original version of this Article contained a typographical error in the Abstract.

“Goraphene derivatives (GD) are currently being evaluated for technological and biomedical applications owing to their unique physico-chemical properties over other carbon allotrope such as carbon nanotubes (CNTs).”

now reads:

“Graphene derivatives (GD) are currently being evaluated for technological and biomedical applications owing to their unique physico-chemical properties over other carbon allotrope such as carbon nanotubes (CNTs).”

This has now been corrected in the PDF and HTML versions of the Article.

