# Author Correction: Physico-chemical properties based differential toxicity of graphene oxide/reduced graphene oxide in human lung cells mediated through oxidative stress

**DOI:** 10.1038/s41598-018-33153-z

**Published:** 2018-10-29

**Authors:** Sandeep Mittal, Veeresh Kumar, Nitesh Dhiman, Lalit Kumar Singh Chauhan, Renu Pasricha, Alok Kumar Pandey

**Affiliations:** 1 Academy of Scientific and Innovative Research (AcSIR), CSIR – IITR Campus, Lucknow, India; 2 Nanomaterials Toxicology Laboratory, Nanotherapeutics and Nanomaterial Toxicology Group, CSIR – Indian Institute of Toxicology Research (CSIR – IITR), Vishvigyan Bhawan, 31, Mahatma Gandhi Marg, PO Box – 80, Lucknow, Uttar Pradesh 226001 India; 3 CSIR - National Physical Laboratory (CSIR-NPL), New Delhi, 110012 India; 4 Water Analysis Laboratory, Nanotherapeutics and Nanomaterial Toxicology Group, CSIR – Indian Institute of Toxicology Research (CSIR – IITR), Vishvigyan Bhawan, 31, Mahatma Gandhi Marg, PO Box – 80, Lucknow, Uttar Pradesh 226001 India; 5 Electron Microscopy Laboratory, CSIR – Indian Institute of Toxicology Research (CSIR – IITR), Vishvigyan Bhawan, 31, Mahatma Gandhi Marg, PO Box – 80, Lucknow, Uttar Pradesh 226001 India

Correction to: *Scientific Reports* 10.1038/srep39548, published online 21 December 2016

This Article contains errors.

In the Abstract,

“This is prior study to the best of our knowledge involving TRGO for its safety evaluation which provided invaluable information and new opportunities for GD based biomedical applications.”

should read:

“This is the first study to the best of our knowledge involving TRGO for its safety evaluation which provided invaluable information and new opportunities for GD based biomedical applications.”

Additionally, in Figure 8, the immunoblots in 8a, 8c, 8e, 8g and 8i were inadvertently misassembled during figure preparation. The correct Figure 8 is shown below as Figure 1.

**Figure 1:**
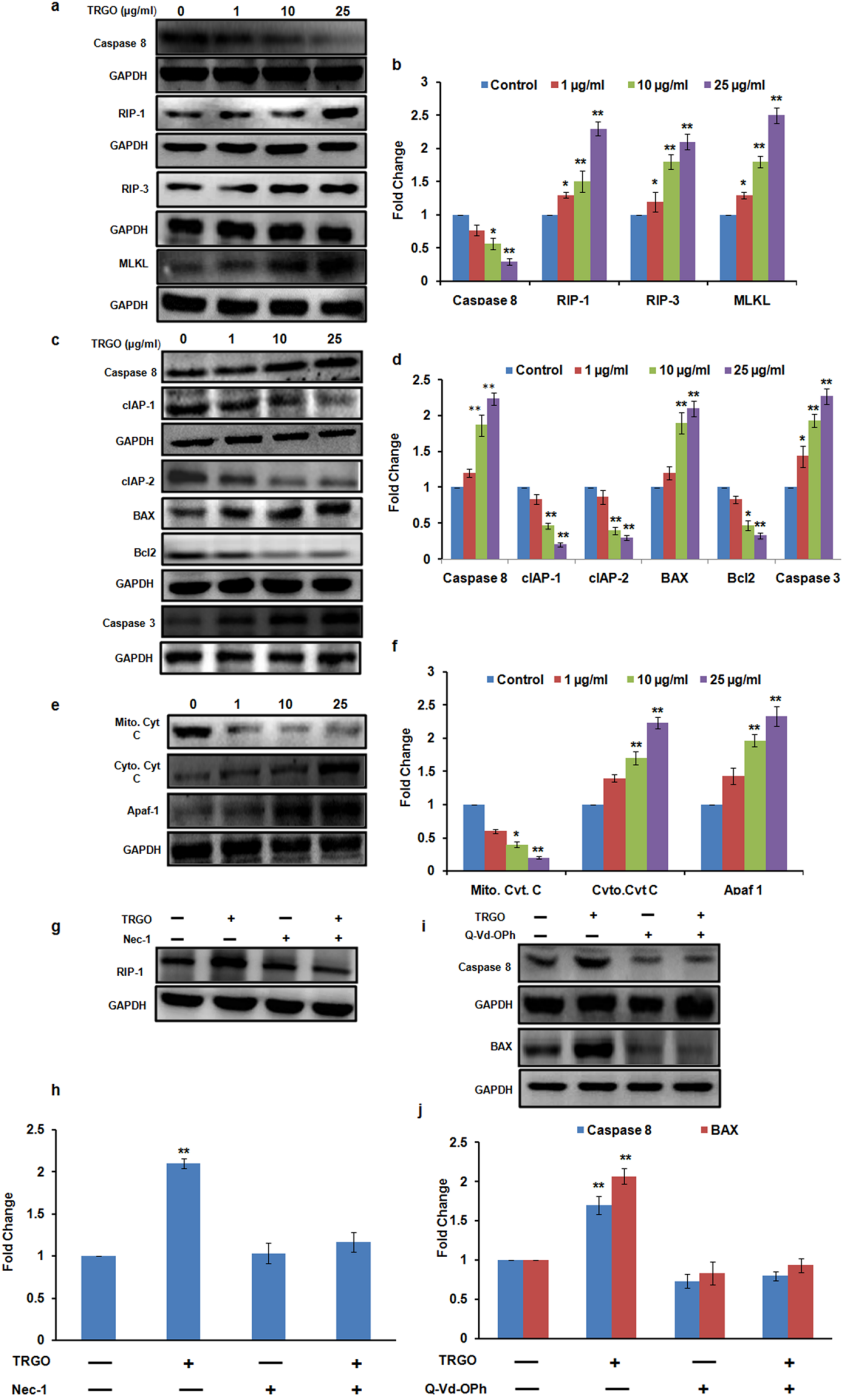
Western blot analysis of GD exposed cells for the evaluation of cell death pathway. A significant increase in the expression level of necroptotic protein in A549 cells (**a**) whereas increase in apoptotic protein in BEAS-2B cells (**c**,**e**) was found after the 24 h exposure of TRGO. (**b**,**d**,**f**) Quantitation was done in Biorad Versa DOC (Bio-Rad Laboratories, Inc. Hercules, USA) with the help of Quantity One Quantitation Software version 4.3.1 and expressed in fold change. Further, in the presence of Nec-1 (**g**) and Q-Vd-OPh (**i**) the expression level of respective proteins was found to get attenuated which confirmed the activation of differential cell death pathway upon GD exposure. (**h**,**j**) Quantitation was done in Biorad Versa DOC (Bio-Rad Laboratories, Inc. Hercules, USA) with the help of Quantity One Quantitation Software version 4.3.1 and expressed in fold change. Full lengths blots were shown in Fig. S11.

The authors did not include corresponding unprocessed full length immunoblots for all cropped figures.

In the updated Supplementary Information file that accompanies this correction: Supplementary Figure S11 has been updated to show the unprocessed full length blots for all immunoblots, including the corrected immunoblots; in the labelling of Figure S7, ‘GO NPs (µg/ml)’, ‘TRGO NPs (µg/ml)’ and ‘CRGO NPs (µg/ml)’ have been updated to ‘GO (µg/ml)’, ‘TRGO (µg/ml)’ and ‘CRGO (µg/ml)’ respectively.

Finally, this Article erroneously contains the following two papers in the reference list.

25. Han, Y. *et al*. Different inhibitory effect and mechanism of hydroxyapatite nanoparticles on normal cells and cancer cells *in vitro* and *in vivo*. *Sci Rep* **4** (2014).

26. Ekstrand-Hammarström, B. *et al*. Human primary bronchial epithelial cells respond differently to titanium dioxide nanoparticles than the lung epithelial cell lines A549 and BEAS-2B. *Nanotoxicology* **6**, 623–634 (2012).

## Electronic supplementary material

Supplementary Information

