# Supplementary material for: Physico-chemical properties based differential toxicity of graphene oxide/reduced graphene oxide in human lung cells mediated through oxidative stress
Source: Sci Rep. 2016 Dec 21;6:39548. doi: 10.1038/srep39548 (PMC5175188; doi:10.1038/srep39548)
Supplement: Supplementary Information [file srep39548-s1.pdf]

Supplementary Information

Physico-chemical properties based differential toxicity of graphene oxide/reduced graphene oxide in human lung cells mediated through oxidative stress

Sandeep Mittal[1,2], Veeresh Kumar[3], Nitesh Dhiman[1,4], Lalit Kumar Singh Chauhan[5], Renu Pasricha[3,*], Alok Kumar Pandey[1,2,*]

Author's Affiliations:

[1]Academy of Scientific and Innovative Research (AcSIR), CSIR – IITR Campus, Lucknow.

[2]Nanomaterials Toxicology Laboratory, Nanotherapeutics and Nanomaterial Toxicology Group, CSIR – Indian Institute of Toxicology Research (CSIR – IITR), Vishvigyan Bhawan, 31, Mahatma Gandhi Marg, PO Box – 80, Lucknow, Uttar Pradesh – 226001.

[3]CSIR – National Physical Laboratory (CSIR-NPL), New Delhi - 110012, India.

[4]Water Analysis Laboratory, Nanotherapeutics and Nanomaterial Toxicology Group, CSIR – Indian Institute of Toxicology Research (CSIR – IITR), Vishvigyan Bhawan, 31, Mahatma Gandhi Marg, PO Box – 80, Lucknow, Uttar Pradesh – 226001.

[5]Electron Microscopy Laboratory, CSIR – Indian Institute of Toxicology Research (CSIR – IITR), Vishvigyan Bhawan, 31, Mahatma Gandhi Marg, PO Box – 80, Lucknow, Uttar Pradesh – 226001

*** Corresponding Authors**

Dr. Alok K. Pandey
Nanotherapeutics and Nanomaterial Toxicology Group
CSIR – Indian Institute of Toxicology Research (CSIR – IITR)
Vishvigyan Bhawan, 31, Mahatma Gandhi Marg, Lucknow – 226001, U.P. (India)
Email: pandeyalok2006@gmail.com, alokpandey@iitr.res.in

Dr. Renu Pasricha
Principal Scientist
CSIR-National Physical Laboratory (CSIR-NPL)
Dr. KS Krishnan Marg, South Patel Nagar
Pusa, New Delhi – 110012, India
Email: renu1505@gmail.com

Methods Section

1. **Cell cycle analysis of GO, TRGO and CRGO exposed A549 and BEAS – 2B cells:** After the completion of exposure, cells were harvested and centrifuged at 1200 rpm for 10 min and the pellet was re-suspended in 1X PBS (300 µl). Cells were fixed with chilled 70% ice cold ethanol and incubated overnight at -20 ^{0}C. Then, cells were centrifuged at 1200 rpm for 4 min and pellet was again suspended in lysis buffer (1X PBS along with 0.2% Triton X-100, 200 µl) and incubated at 4 ^{0}C for 30 min. Lysed cells were centrifuged at 1200 rpm for 10 min and pellet was resuspended in 1X PBS (1 ml) containing RNase (10 mg/ml, 20 µl) and incubated for 30 min at 37 ^{0}C. Cells were again centrifuged at 1200 rpm for 10 min and pellet was resuspended in 1X PBS (500 µl) containing Propidium iodide dye (1 mg/ml, 10 µl) and stored at 4 ^{0}C until read at flow cytometer (FACS Canto^{TM} II, BD BioSciences, San Jose, CA, USA).
2. **Genotoxicity potential of GO, TRGO and CRGO:** Briefly, cells were cultured at a density of 2 x 10^5 cells/ml/well in 6 well plate, exposed to GD for 3 h and 6 h. After that, cells were washed and incubated with fresh growth medium till next division cycle. Upon completion, cells were harvested, re-suspended in solution I (10 mM NaCl, 3.4 mM sodium citrate, 10 mg/L RNase, 0.3 mg/L IGEPAL and 25 mg/L ethidium bromide (EtBr) and incubated for 45 min at room temperature. Then equal volume of solution II (1.5% citric acid, 0.25 M sucrose, 40 mg/L EtBr) was added and samples were read at flow cytometer (FACS Canto^{TM} II, BD BioSciences, San Jose, CA, USA). Results were expressed as percentage micronuclei formed per nucleus.
3. **Western Blot Analysis:** Exposed cells were washed, scraped in 1X PBS and centrifuged at 1000 rpm for 10 min. Cell pellet were suspended in ice cold cell lytic buffer containing protease inhibitor cocktail and sonicated for 20 sec with 5 sec pulse on and 10 sec pulse off using probe sonicator. The prepared homogenate were centrifuged at 15000 rpm for

30 min at 4 ^{0}C to obtain supernatant. The protein content of supernatant was measured using BCA kit (Genei™, Merck Millipore Pvt Ltd., Mumbai). Equal amount of protein was separated on sodium dodecyl sulfate-polyacrylamide gels (SDS-PAGE) and transferred to polyvinylidene fluoride (PVDF) membrane. The membrane were blocked using blocking buffer (Sigma-Aldrich, St. Louis, USA) for 1 h at room temperature and then incubated with anti-BAX, anti-Bcl-2, anti-caspase-3, anti-caspase-9, anti-Apaf-1, anti-cytochrome-C, anti-RIP-1, anti-RIP-3, anti-MLKL and anti-GAPDH for overnight at 4 ^{0}C temperature. Blots were further incubated with horseradish peroxidase conjugated secondary antibody for 2 h at room temperature and proteins were visualized using Super Signal West Femto Maximum Sensitivity Substrate Solution (Thermo Fisher Scientific, Waltham, USA) on Biorad Versa DOC (Bio-Rad Laboratories, Inc. Hercules, USA). Densitometric analysis was done using Quantity One Quantitation Software version 4.3.1(Bio-Rad Laboratories, Inc. Hercules, USA).

Results Section

1. Synthesis of graphene derivatives:

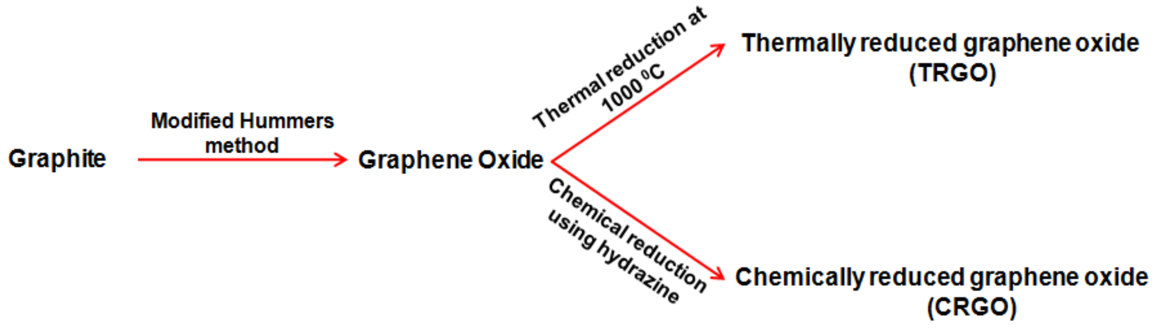

Figure S1. Schematic representation showing the synthesis of graphene oxide (GO), thermally reduced graphene oxide (TRGO) and chemically reduced graphene oxide (CRGO) via the oxidation and reduction of graphite.

2. Characterization of graphene derivatives:

Table S1: Dynamic light scattering analysis of GO, TRGO and CRGO for their hydrodynamic size and stability in culture medium.

Graphene Derivatives	Hydrodynamic size in culture medium (d.nm)	Zeta Potential in Milli-Q water (mV)	Zeta Potential in culture medium (mV)
Graphene Oxide (GO)	126.3	-30.48	-13.7
Thermally reduced graphene oxide (TRGO)	66.98	-9.5	-6.90
Chemically reduced graphene oxide (CRGO)	97.3	-18.12	-9.46

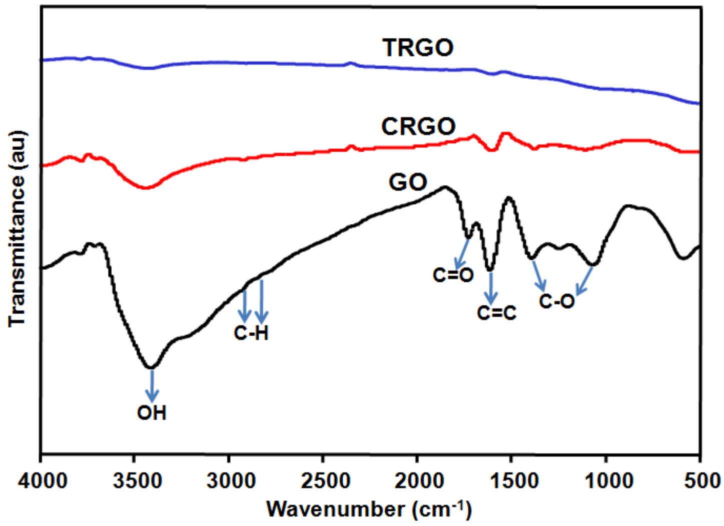

Figure S2. Fourier transforms infrared spectroscopy (FTIR) analysis. The presence of functional group analysis was carried out using FTIR which demonstrate the oxygen containing functional group on the surface of GO which was found to get reduce after their thermal reduction (TRGO) as well as chemical reduction (CRGO).

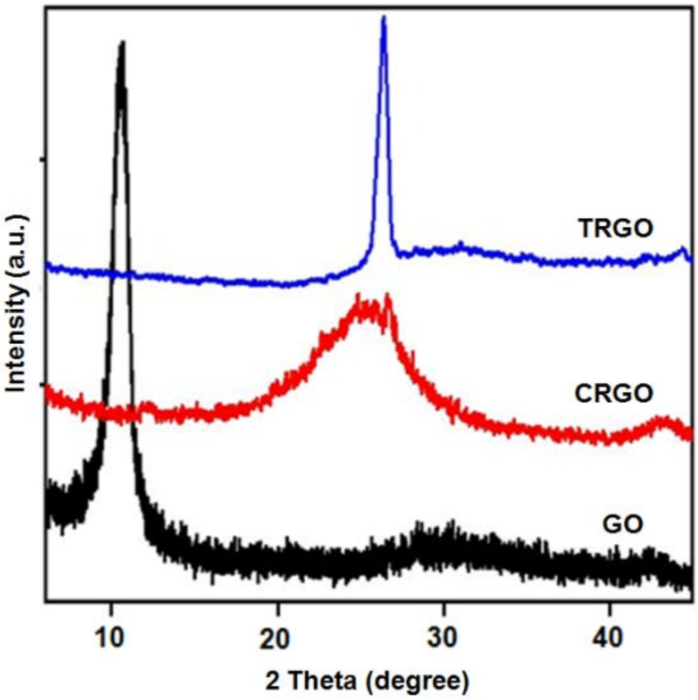

Figure S3. X-Ray diffraction spectroscopy (XRD). Phase identification analysis of GO, TRGO and CRGO.

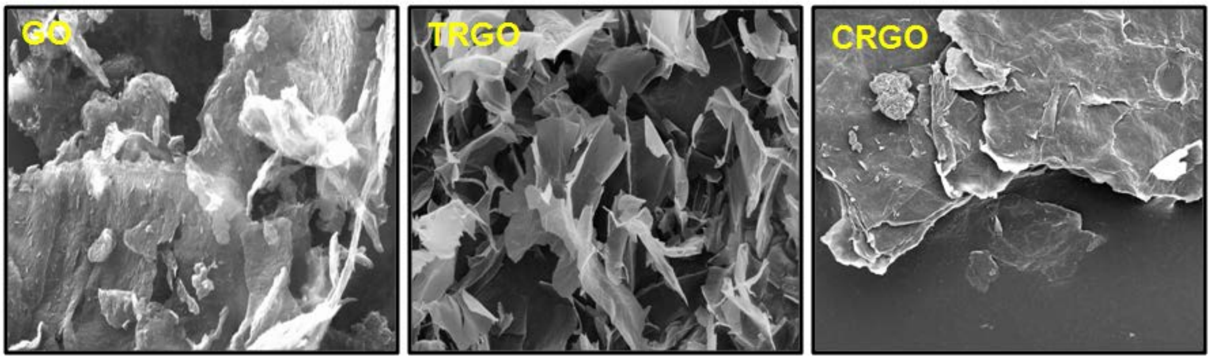

Figure S4. Scanning electron micrographs (SEM). The surface morphology of (A) GO (B) TRGO and (C) CRGO.

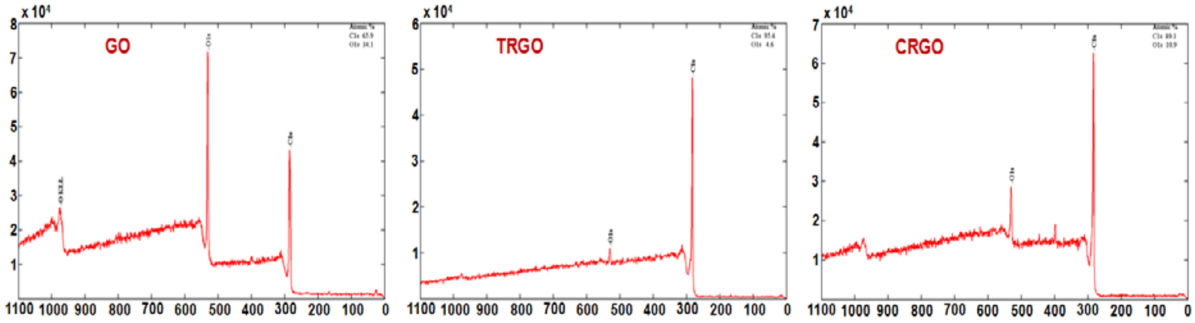

Figure S5. X-ray photoelectron spectroscopy (XPS). Survey scan spectra of GO, TRGO and CRGO showing the percentage of carbon and oxygen.

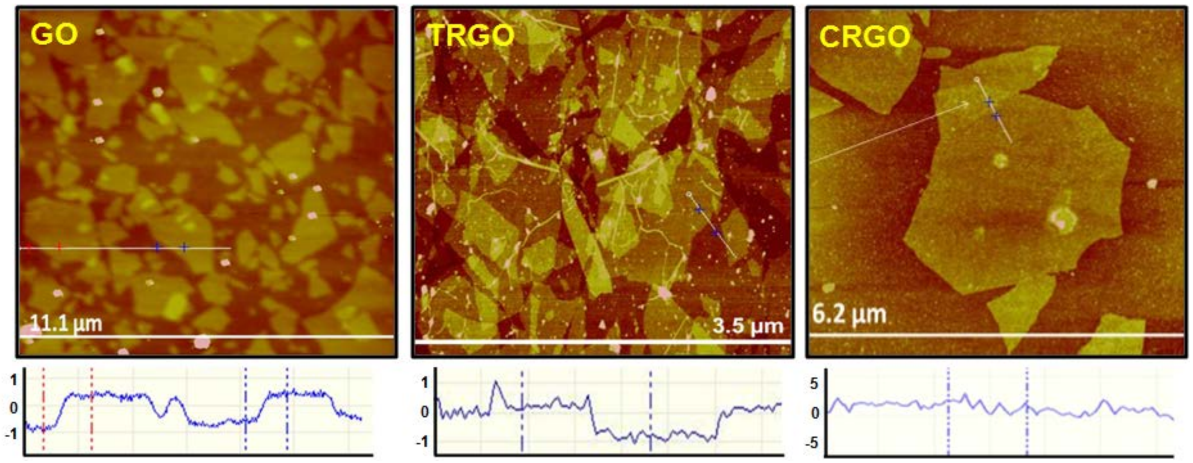

Figure S6. Atomic force microscopy (AFM) analysis for the height and thickness profile of GO, TRGO and CRGO.

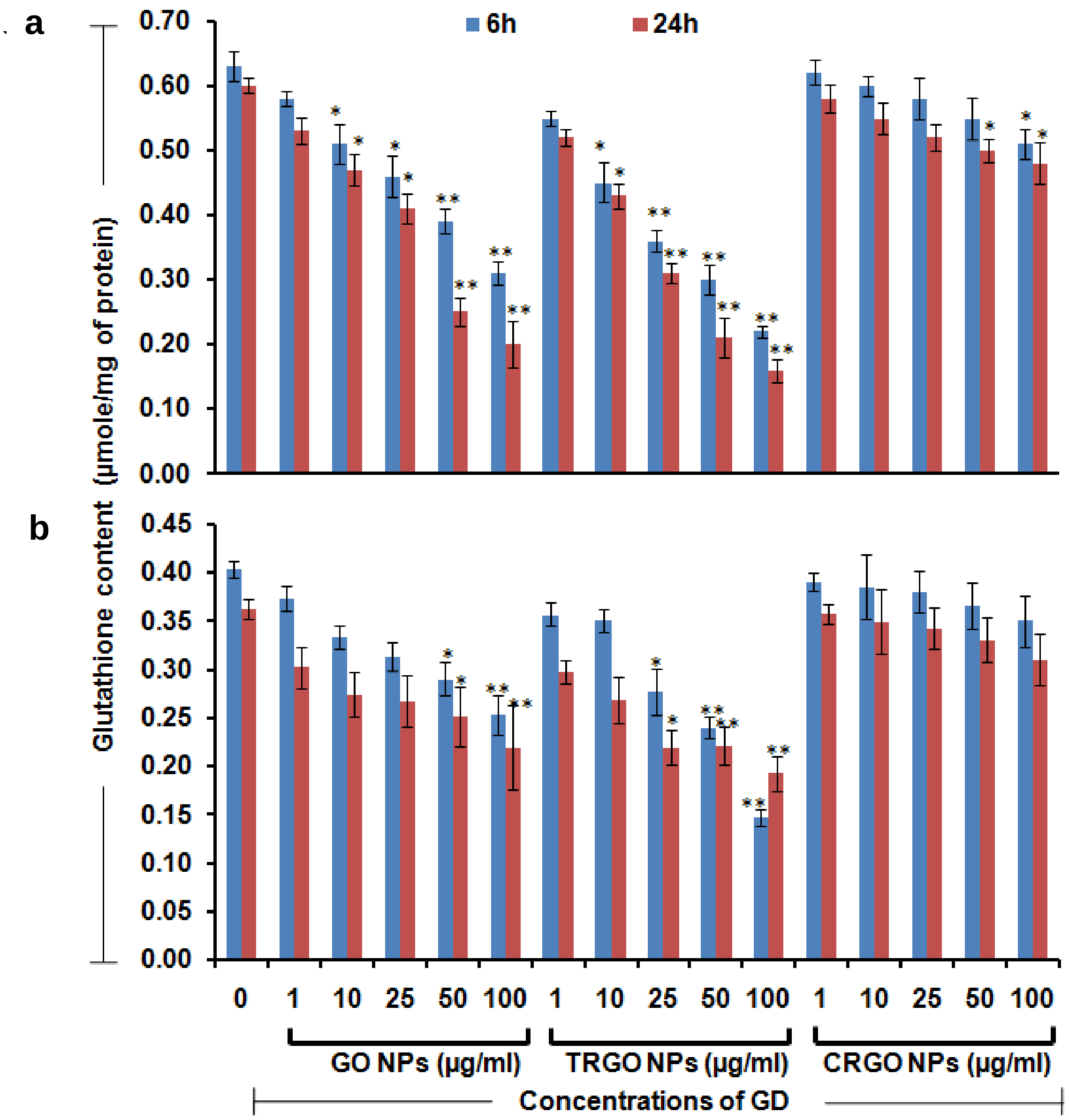

Figure S7. Oxidative stress measurement. Cellular glutathione depletion of (a) A549 cells (b) BEAS-2B cells was analyzed as a marker of oxidative stress induction after 6 h and 24h exposure of GO, TRGO and CRGO. Data represents mean ± SE of three independent experiment. *p < 0.05, 0.01 when compared with control group.

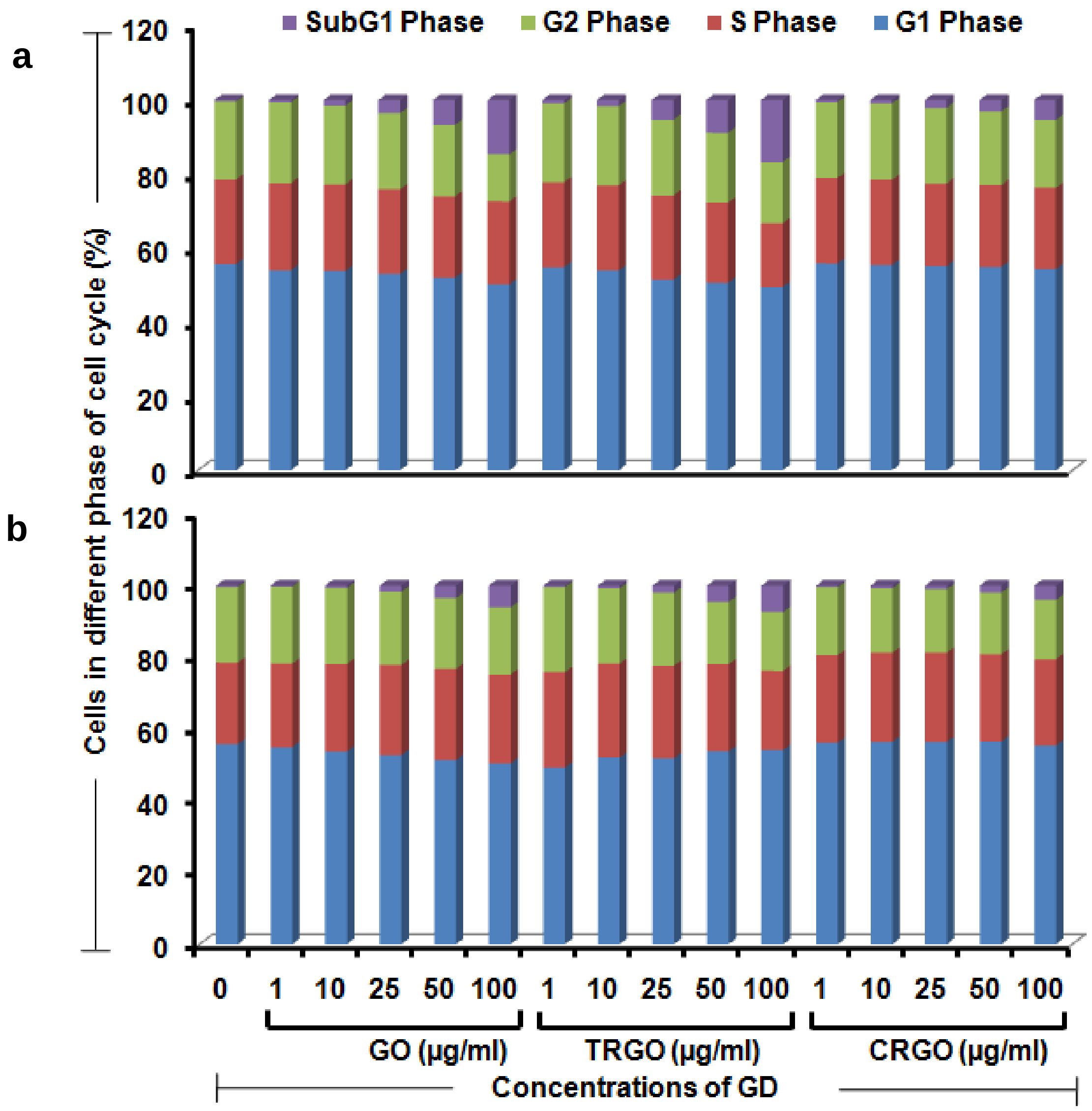

Figure S8. Cell cycle progression analysis. Perturbation in cell cycle progression of (a) A549 cells (b) BEAS-2B cells exposed to GO, TRGO and CRGO for 24 h was analyzed using flow cytometry based propidium iodide method.

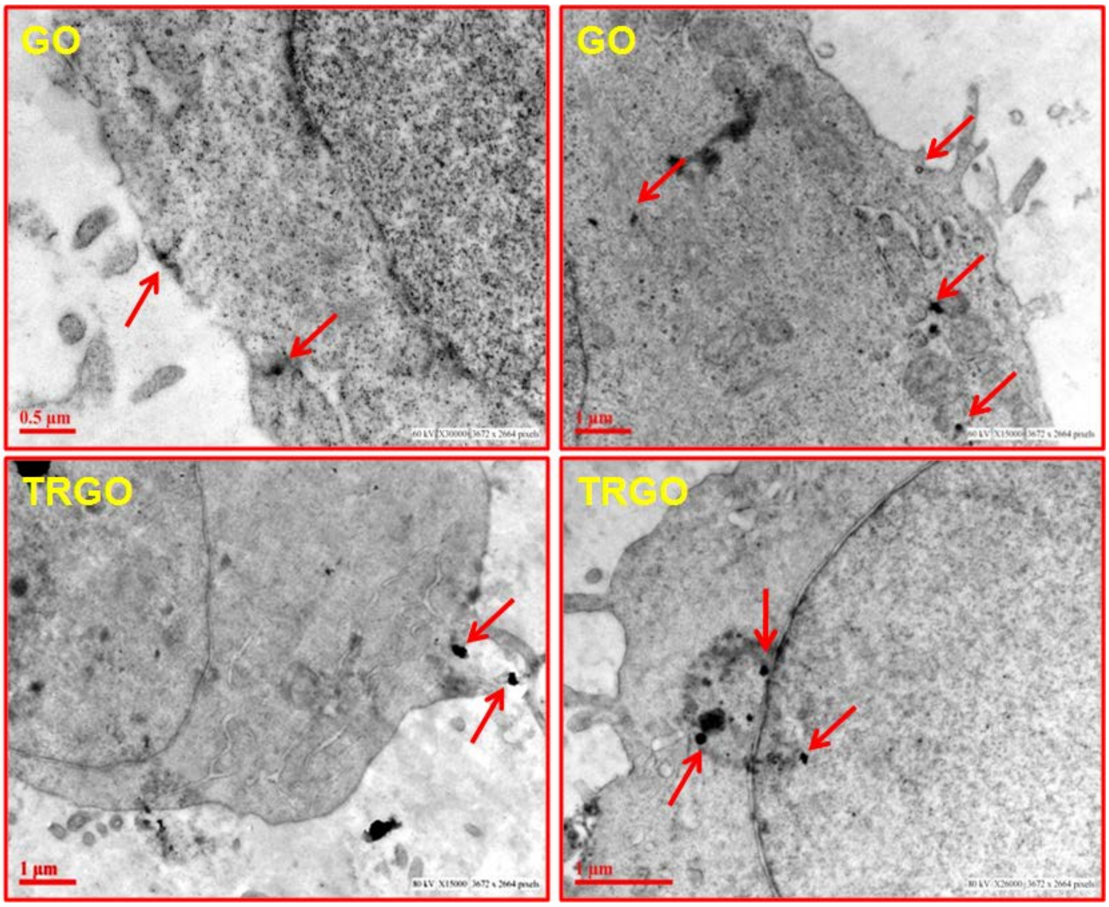

Figure S9. Cellular internalization mechanism of GD. TEM photomicrographs showing the different internalization mechanism for GO and TRGO in A549 cells. GO parallel aligned to the membrane and get internalize by passive mechanism whereas TRGO owing to their small size efficiently taken up by cells and reached to the nucleus (red arrow showing the presence of GD).

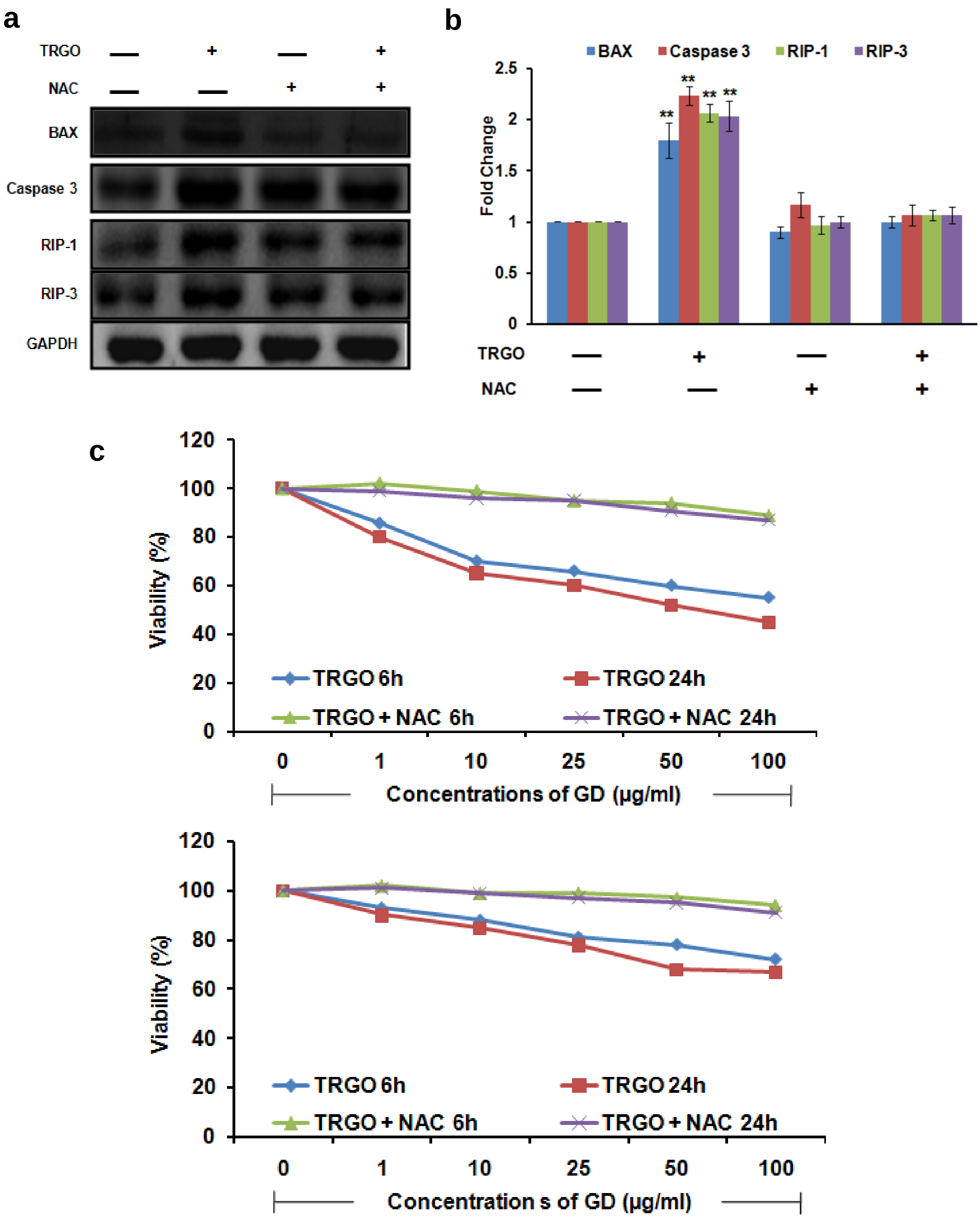

Figure S10. Effect of NAC on cell death mechanism and cellular viability. (a) The pretreatment of NAC prior to GD exposure down regulate the expression level of necroptosis as well as apoptotic protein in A549 and BEAS-2B cells. (b) Quantitation was done in Biorad Versa DOC (Bio-Rad Laboratories, Inc. Hercules, USA) with the help of Quantity One

Quantitation Software version 4.3.1 and expressed in fold change. (c) Including this, NAC also attenuate the reduction in viability of A549 cells (c-upper panel) and BEAS-2B (c-lower panel) upon the 6 h and 24 h exposure of TRGO.

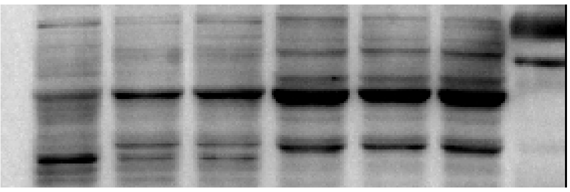

Original image for RIP1 (Fig. 8a)

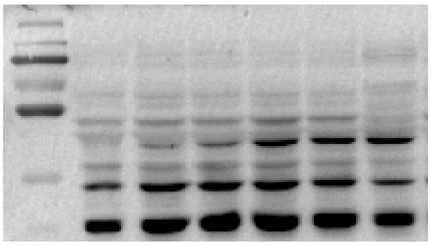

Original image for MLKL (Fig. 8a)

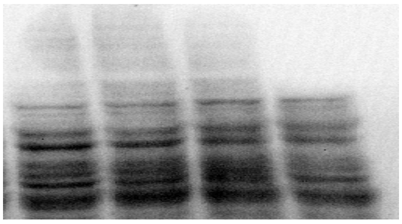

Original image for cIAP1 (Fig. 8c)

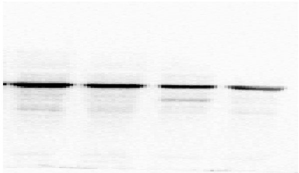

Original image for Bcl2 (Fig. 8c)

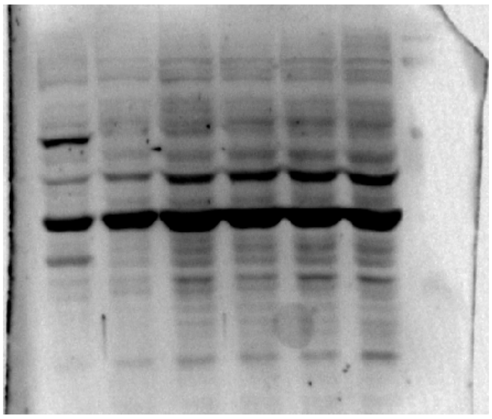

Original image for RIP3 (Fig. 8a)

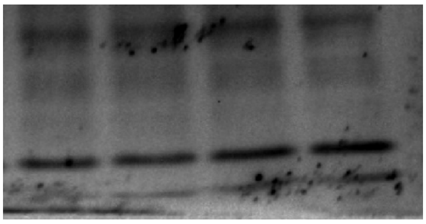

Original image for caspase 8 (Fig. 8c)

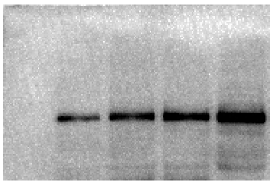

Original image for BAX (Fig. 8c)

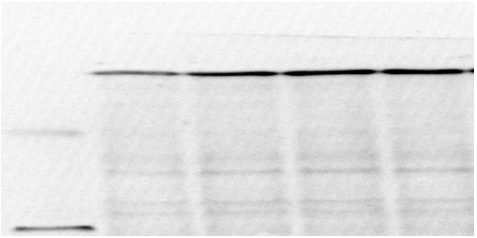

Original image for Mito Cyt C (Fig. 8c)

Figure S11. Full blot images.
